# The systemic tumor response to RNase A treatment affects the expression of genes involved in maintaining cell malignancy

**DOI:** 10.18632/oncotarget.20228

**Published:** 2017-08-12

**Authors:** Nadezhda Mironova, Olga Patutina, Evgenyi Brenner, Alexander Kurilshikov, Valentin Vlassov, Marina Zenkova

**Affiliations:** ^1^ Institute of Chemical Biology and Fundamental Medicine SB RAS, Novosibirsk, Russia; ^2^ Department of Genetics, University Medical Center Groningen, University of Groningen, Groningen, The Netherlands

**Keywords:** antitumor ribonucleases, RNase A, sequencing, metabolism of cancer cells, cancer-related pathways

## Abstract

Recently, pancreatic RNase A was shown to inhibit tumor and metastasis growth that accompanied by global alteration of miRNA profiles in the blood and tumor tissue (Mironova et al., 2013). Here, we performed a whole transcriptome analysis of murine Lewis lung carcinoma (LLC) after treatment of tumor-bearing mice with RNase A. We identified 966 differentially expressed transcripts in LLC tumors, of which 322 were upregulated and 644 were downregulated after RNase A treatment. Many of these genes are involved in signaling pathways that regulate energy metabolism, cell-growth promoting and transforming activity, modulation of the cancer microenvironment and extracellular matrix components, and cellular proliferation and differentiation. Following RNase A treatment, we detected an upregulation of carbohydrate metabolism, inositol phosphate cascade and oxidative phosphorylation, re-arrangement of cell adhesion, cell cycle control, apoptosis, and transcription. Whereas cancer-related signaling pathways (e.g., TGF-beta, JAK/STAT, and Wnt) were downregulated following RNase A treatment, as in the case of the PI3K/AKT pathway, which is involved in the progression of non-small lung cancer. RNase A therapy resulted in the downregulation of genes that inhibit the biogenesis of some miRNAs, particularly the let-7 miRNA family.

Taken together, our data suggest that the antitumor activity and decreased invasion potential of tumor cells caused by RNase A are associated with enhanced energy cascade functioning, rearrangement of cancer-related events regulating cell growth and dissemination, and attenuation of signaling pathways having tumor-promoting activity. Thus, RNase A can be proposed as a potential component of anticancer therapy with multiple modes of action.

## INTRODUCTION

Over the past three decades, remarkable progress has been made in clarifying the mechanisms of cancer pathogenesis, encouraging the development of novel therapeutic strategies. Tumor development is accompanied by a variety of disorders, such as fast unlimited proliferation, resistance to tumor suppressors, loss of the initial differentiation, cell death resistance, replicative immortality, reprogramming of energy metabolism, evasion from immune surveillance, induction of angiogenesis, infiltrated growth and dissemination [1]. Chemotherapy remains the standard cancer treatment but is limited to universal impact on the proliferative properties of the tumor, which results in a loss of efficiency in some cases. Work addressing the properties and functioning of tumor cells has enabled the development of targeted anticancer drugs, focused on those mechanisms necessary for tumor growth and progression. Over the last decade, the introduction of targeted therapies into clinical practice has increased the survival rate of cancer patients. Nevertheless, the most important feature of malignant disease is the deployment of regulatory cascades directed to maintaining tumor cell survival, thus enabling the damaged cells to adapt to a selective pressure and elude narrowly focused therapeutic effects. The complexity of the process of malignant transformation is enhanced by the fact that tumor is capable of recruiting the neighboring normal cells that form a tumor microenvironment and enhance tumor progression [1]. As a result, the tumor is a heterogeneous tissue containing regions of various degrees of differentiation, vascularity, migratory potential and, thus, possessing complicated intra- and extra-cellular regulation. Thus, to overcome the heterogeneity, dynamism of molecular profile, and flexibility of regulation necessary for the survival of tumor cells, requires the application of drugs of multifactor action. The impact on the regulatory system of the tumor, controlling simultaneously multiple signaling pathways, can allow preventing the development of adaptive resistance of the tumor and appears to be an effective approach to struggle with neoplasia.

A rapidly growing amount of data indicates that miRNAs have a significant regulatory role in the vital activity of tumor cells [2, 3]. Hundreds of distinct miRNAs interact with target genes and operate as an important part of a large regulatory network, promoting tumorigenesis [2, 3]. Thus, ribonucleases, enzymes that can damage RNA and inhibit RNA-dependent regulatory processes in the tumor, represent promising alternative to conventional DNA-damaging agents and targeted drugs. It has been shown that the application of RNases as antitumor therapeutics helps to counterbalance the pathological molecular changes that occur within tumor cells and, thus, to control their malignant behavior. The antitumor and antimetastatic activity, as well as general system effects, of natural RNases have been demonstrated (e.g., BS-RNase from bovine testis [4-7], onconase from oocytes of *Rana pipiens* [8-10], bovine pancreatic RNase A [11-13], and microbial RNases [14-17]). The antitumor effects of Onconase [18, 19] and RNase A [20] were shown to be associated with an alteration in miRNA profiles in tumor cells. In malignant pleural mesothelioma cell lines, onconase was shown to significantly upregulate hsa-miR-17 and downregulate hsa-mir-30c, resulting in NFk-β inhibition and an increase in the chemosensitivity of tumor cells [18]. Onconase also downregulates intracellular miRNAs by cleaving miRNA precursors in Msto-211h mesothelioma cells [19].

Recently, using two murine tumor models, our group has shown that the pancreatic RNase A is capable of retarding primary tumor growth and efficiently inhibiting the development of metastases [11-13]. Using a murine Lewis lung carcinoma (LLC) model, we examined tumor and blood miRNA profiles after RNase A treatment and found that the antitumor and antimetastatic activities of the enzyme were associated with an increase in tumor miRNAs and decrease in serum miRNAs [20]. Because of the regulatory role of miRNAs, here we performed a whole transcriptome analysis of LLC following RNase A treatment by SOLiD™ sequencing. By this approach, we were able to identify key genes and event cascades crucial for the tumoricidal and invasive properties of tumor cells and, thus, explain the observed antitumor activity of the enzyme.

## RESULTS

### Design of the experiment and sequencing

RNase A treatment was shown to alter the tumor and blood miRNA profiles of LLC-bearing mice [20], decreasing the invasive potential of tumor cells and metastasis spreading [11-13]. Because there is still no evidence that RNase A penetrates into tumor cells or evades cytosolic ribonuclease inhibitor RI, it should be assumed that the observed effect is a systemic response of the tumor to treatment. Here, by SOLiD™ sequencing, we performed whole transcriptome analysis of LLC after treatment with RNase A and attempted to identify changes in the expression of key tumor survival cells.

**Figure 1:**
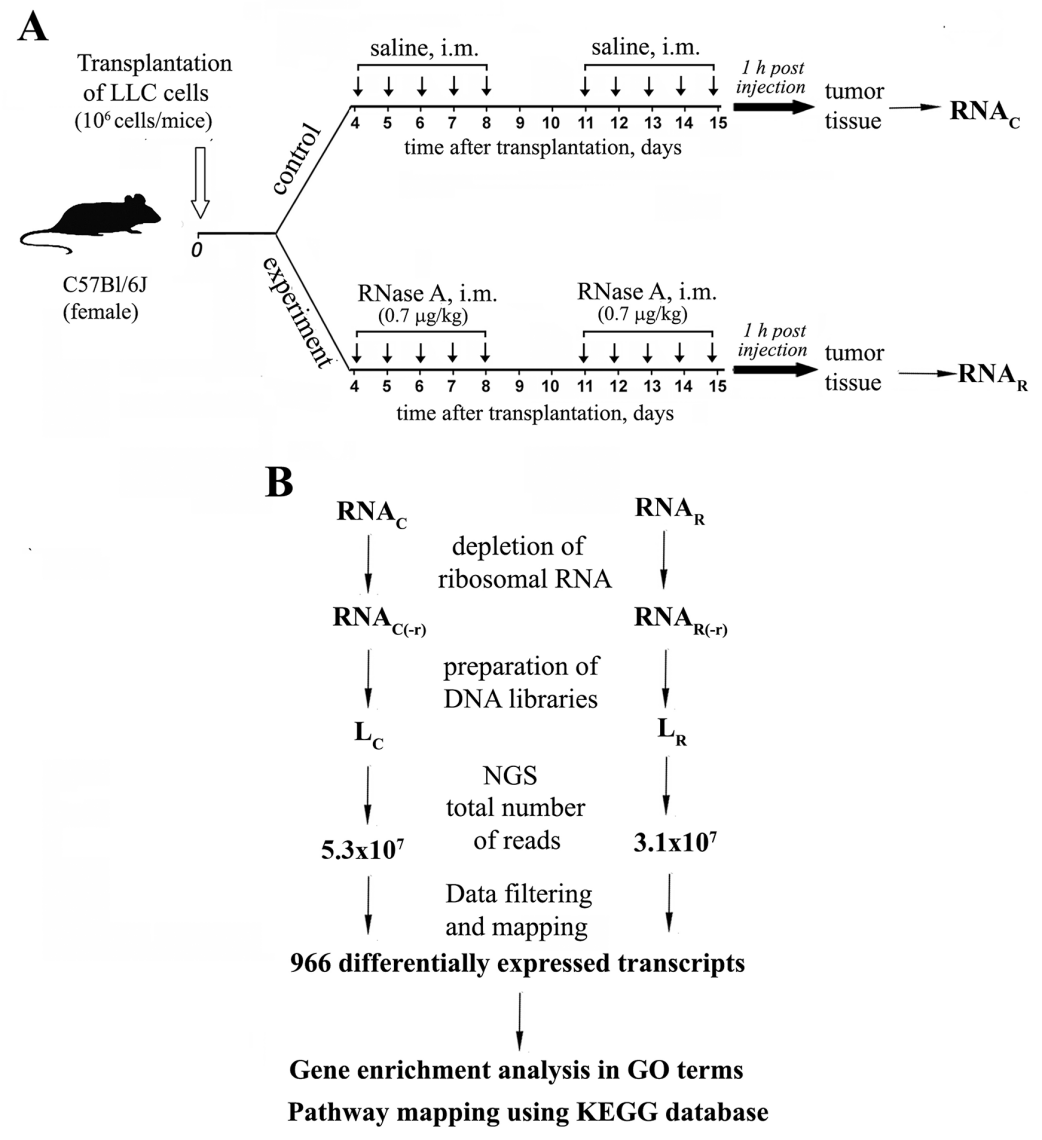
Experimental design and data mining **(A)** Mice with i.m. transplanted LLC were treated with saline or RNase A at a dose of 0.7 μg/kg for 10 days starting on the 4^th^ day after tumor transplantation. At 1 h after the last injection, tumor tissue samples were collected. **(B)** Total RNA was isolated and pooled according to groups. mRNA fractions were enriched by ribosomal RNA depletion and used for the construction of cDNA libraries. Libraries were sequenced using the standard SOLiD™ V5.5 (Applied Biosystems) protocols. Reads were mapped to the *Mus musculus* reference genome (version NCBI37), and analysis of differential expression was performed. Differentially expressed transcripts were annotated to GO terms and analyzed using KEGG database to assign pathway mapping.

A scheme of the experiment is shown in Figure 1. Following on protocol from our previous work [13, 20], two groups of mice with intramuscularly (i.m.) implanted LLC were treated with either saline buffer or RNase A. On the 15^th^-day post LLC transplantation and after ten injections of RNase A, tumor samples were collected and pooled according to groups. Total RNA was isolated and depleted of rRNAs. L_C_ (saline treated) and L_R_ (RNase A treated) cDNA libraries were prepared and sequenced using a SOLiD™ ABA 5.5 platform. The total numbers of reads for the L_C_ and L_R_ libraries were 5.3 × 10^7^ and 3.1 × 10^7^, respectively.

### Gene annotation

**Figure 2:**
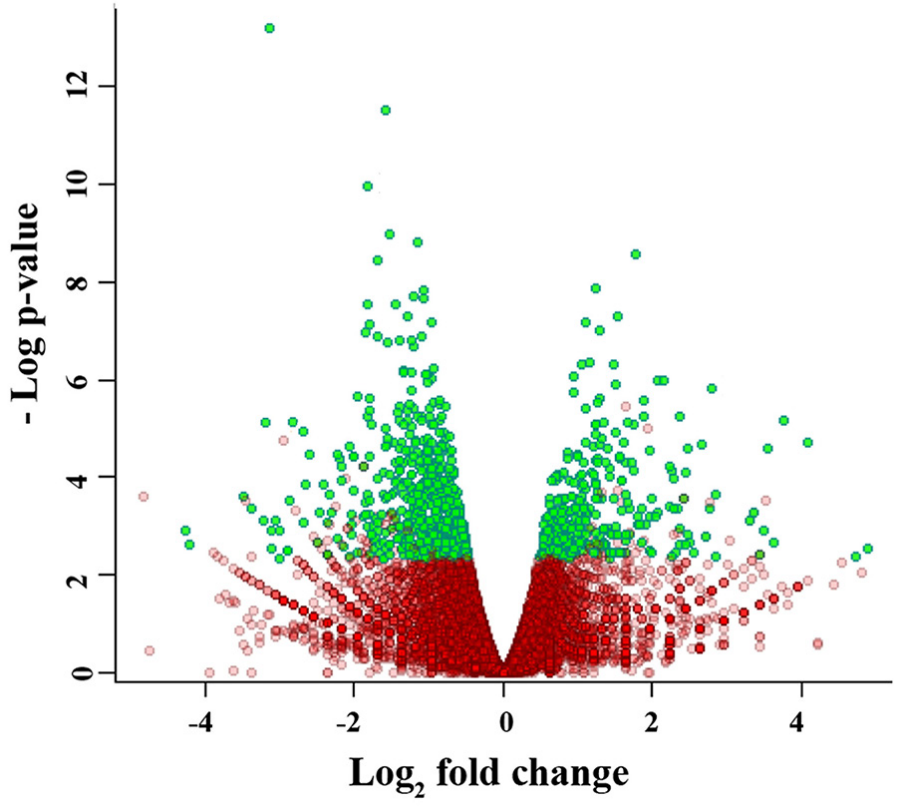
Volcano plot of the gene expression data Green dots represent the genes for which transcription level was significantly different between samples of treated and untreated mice.

Reads were mapped to the *Mus musculus* reference genome (version NCBI37) using Bioscope software v.1.3. (ABI, USA). The differential expression analysis revealed 966 differentially expressed transcripts (qFDR < 0.05), of which 322 were upregulated and 644 were downregulated in the tumor tissue of the L_R_ group, compared to the L_C_ group (Figure 2).

**Figure 3:**
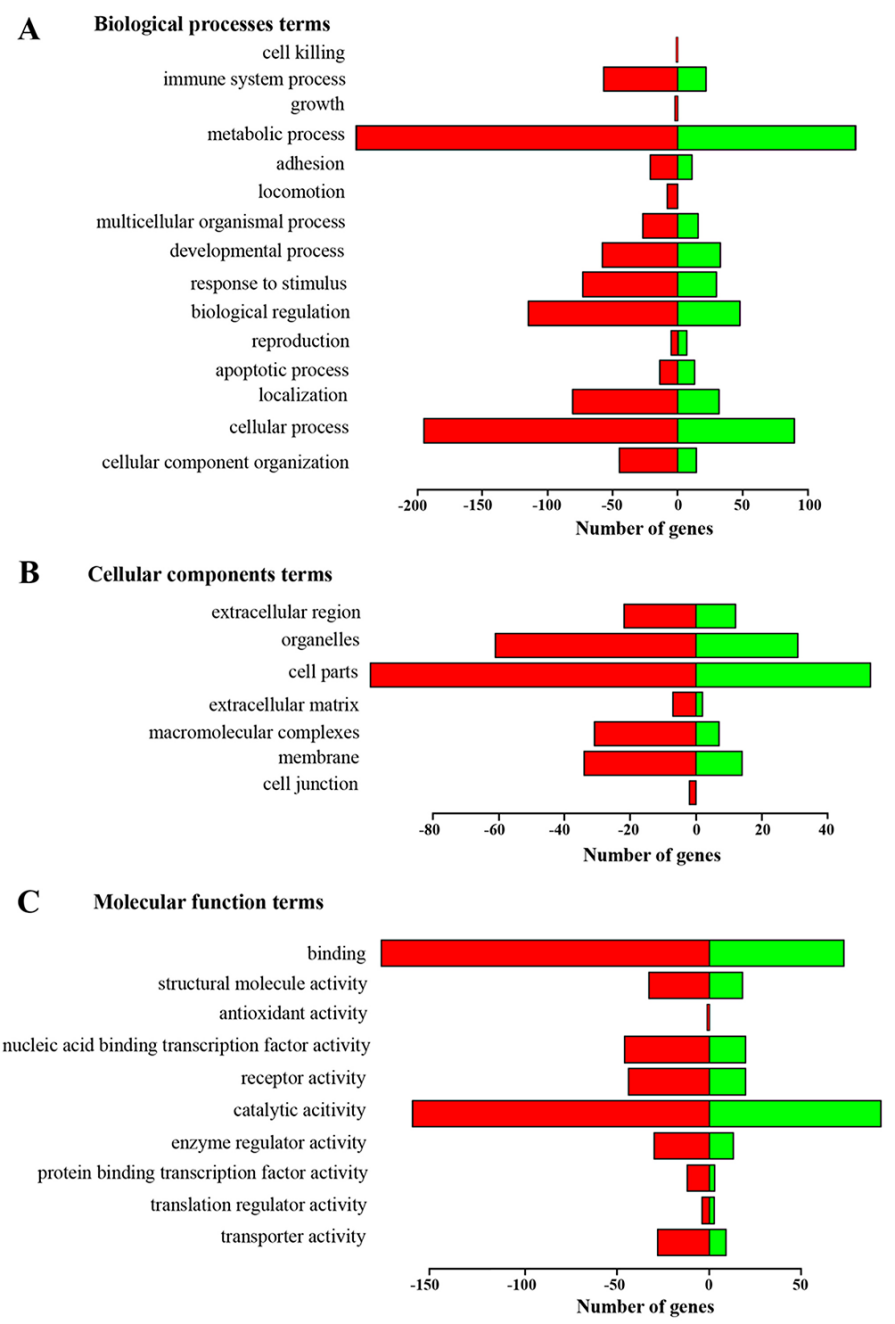
Functional annotation of genes changed more than 1.4-fold in tumor tissue of LLC-bearing mice after treatment with RNase A based on gene ontology (GO) categorization **(A)** Biological Process [BP]. **(B)** Molecular Function [MF]. **(C)** Cellular Components [CC]. The green bars show the number of genes enriched in the sample of treated mice; red bars show the number of genes with decreased transcription in a group of treated mice.

By gene ontology (GO) analysis (Biological Process [BP], Molecular Function [MF] and Cellular Components [CC]), we found that, for BP, most of the significant changes in expression were observed in genes involved in metabolic and cellular processes (GO:0008152 and GO:0009987), biological regulation (GO:0065007), and response to stimulus (GO:0050896) (Figure 3). For MF, catalytic activity (GO:0003824) and binding (GO:0005488) were the most regulated terms. For CC, the major changes were observed in GO:0044464 (cell part) and GO:0043226 (organelle) (Figure 3).

### Analysis of sequencing data

We used the online KEGG Automatic Annotation Server to assign the obtained gene sequences to those metabolic pathways with significance for cancer progression and cancer-related pathways promoting cell growth and transformation. Data were also analyzed using the Gene Card database. By this approach, we were able to identify tumor inductors and suppressors and miRNA-associated genes, as well as transcription activators, repressors, regulators, and transcription factors (Table 1 and Supplementary Tables 1 –4). In our analysis we took into account a number of up- and downregulated genes in L_R_ in comparison with L_C_ and their involvement in regulation of pathways being important for survival of tumor cell.

**Table 1:** Pathways affected by RNase A in tumor cells

Pathway or event	Up-regulated^a^	Down-regulated^b^	p-Value^c^
**Metabolic pathways**			
Amino sugar and nucleotide sugar metabolism, amino acids and nucleotide metabolism	**4:** *Uap1l1*, *Mtap*, *Cant1*, *Impdh2*	**8:** *Nos2*, *Dguok*, *Adprm*, *Twistnb*, *Setdb2*, *Ha*l, *Amdhd1*, *Gnpnat1*	9.373E-4
Carbohydrate metabolism	**9:** *Pgls*, *Gaa*, *Bpgm*, *Mcee*, *B4galt2*, *Xylb*, *Pdk4*, *Aldoc*, *Lalba*	**8:** *Eno3*, *Galnt3*, *Alg10b*, *Csgalnact2*, *Alg6*, *Ndst2*, *Gxylt1*, *Xylt1*	1.427E-13
Cytochrome P450-associated metabolism	**1:** *Akr7a5*	**3:** *Hsd11b1*, *Cyp27b1*, *Cyp26b1*	2.075E-4
Inositol phosphate	**5:** *Impa1*, *Gpaa1*, *Ip6k2*, *Plcd1*, *Pigb*	**1:** *Pi4k2b*	
Metabolism of lipids and fatty acids	**11:** *Ech1*, *Hadh*, *Eci2*, *Acadvl*, *Pld3*, *Cbr4*, *Acat2*, *Phospho1*, *Plcxd2*, *Acsbg1*, *St3gal5*	**7:** *Agpat2*, *Cd5l*, *Mcat*, *Elovl6*, *Lipt1*, *B4galt6*, *B3galnt1*	2.848E-11
Oxidative phosphorylation	**6:** *Ndufv3*, *Cox8a*, *Foxred1*, *Ndufb11*, *Bcs1l*, *Cox18*	**3:** *Cox7b*, *Ndufa5*, *Atp8b4*	7.042E-9
Nicotinate and nicotinamide metabolism	**2:** *Nmnat3*, *Art5*	**1:** *Bst1*	5.239E-6
Glutathione metabolism	**2:** *Chac1*, *Haghl*	**3:** *Gclm*, *Gss*, *Gpx7*	2.322E-7
**Cancer-related events**			
Angiogenesis	**2:** *Adamts10*, *Robo4*	**4:** *Cxcl5*, *Filip1l*, *Smoc2*, *Angptl1*	- ^d^
Apoptosis	**8:** *Pcbp4*, *Steap3*, *Ctsh*, *Faim*, *Pycard*, *Plekhf1*, *Nol3*, *Dapk1*	**12:** *Lcn2*, *Ctsc*, *Gzmb*, *Hipk3*, *Phlda1*, *Bcl2l2*, *Casp9*, *Ctso*, *Casp12*, *Coro2a*	4.643E-9
Cell adhesion, migration, invasion	**8:** *Rap2a*, *Myl12b*, *Emp2*, *Ptpn14*, *Bcas3*, *Abi3*, *Ajap1*, *Jup*	**12:** *S100a4*, *Col1a1*, *Cav2*, *Rab1*, *Thbs1*, *Rasgrf1*, *Shc4*, *Lrg1*, *Cxcl5*, *Itga7*, *Smoc2*, *Ccnd2*	1.024E-11
Cell cycle control, transformation	**4:** *Usp10*, *Mad2l2*, *Cdc26*, *Rnf122*	**5:** *S100a9*, *Nedd8*, *Incenp*, *Cenpw*, *Nudt6*	2.000E-3
**Cancer-related signaling pathways**			
PI3K/AKT signaling pathway**	**3:** *Angpt2*, *Il2rb*, *Hsp90aa1*	**12:** *Col1a1*, *Thbs1*, *Il4ra*, *Csf3*, *Jak2*, *Tnc*, *Jak3*, *Itga7*, *Csf3r*, *Il7r*, *Ccnd2*, *Itga4*	2.827E-24
RAS signaling pathway	**4:** *Rras*, *Grap*, *Angpt2*, *Rgs14*	**4:** *Rasgrf1*, *Kras*, *Shc4*, *Pld1*	9.345E-10
MAPK signaling pathway	**6:** *Rras*, *Trib3*, *Map3k6*, *Hspa1b*, *Lamtor1*, *Dok4*	**11:** Ccl7, Dusp6, Il1b, Cd14, *Rasgrf1*, *Stk24*, *Il1r2*, *Ccl5*, *Map2k4*, *Hspa2*, *Itgax*	1.869E-13
TGF-β signaling pathway	**2:** *Fam89b*, *Fmod*	**6:** *Ccl7*, *Thbs1*, *Ccl5*, *Acvr1b*, *Rbx1*, *Fst*	2.863E-8
Wnt signaling pathway	**5:** *Serpinf1*, *Wnt8a*, *Vangl1*, *Bcl9*, *Shisa2*	**7:** *Chd8*, *Lrrfip2*, *Dkk2*, *Hmgxb4*, *Rbx1*, *Lgr4*, *Ccnd2*	8.037E-13
JAK-STAT pathway	**2**: *Il12rb1*, *Il2rb*	**11:** *Ccl7*, *Il4ra*, *Shcbp1*, *Ccl5*, *Csf3*, *Jak2*, *Jak3*, *Csf3r*, *Il21r*, *Il7r*, *Ccnd2*	4.607E-18
Calcium signaling pathway	**3:** *Tnnc2*, *Camk2g*, *Tnnc1*	**2:** *P2rx7*, *Tnc*	
**MicroRNA in cancer**	-	**5**: *Ezh2*, *Lin28a*, *Zcchc6*, *Tnrc6a*, *Zcchc11*	8.498E-7
**Cancer-associated**	-	**12:** *Orai1*, *Dpp3*, *Arhgef1*, *Steap1*, *Arhgef11*, *Skp2*, *Tpd52*, *Mllt11*, *Laptm4b*, *Rfng*, *Ehbp1*, *Rbm6*	- ^d^
**Tumor suppressors**	**3:** *Cyb561d2*, *Trit1*, *Pdgfrl*	**4**: *Armcx1*, *Brca2*, *Tssc1*, *Scai*	- ^d^

^a^Data are presented in Supplementary Table 1.

^b^Data are presented in Supplementary Table 2.

^c^p-Value was calculated using tool for transcriptome annotation based gene list functional enrichment analysis ToppFun (https://toppgene.cchmc.org).

^d^Genes belonging to biological process on the base of Gene Card data.

Genes changed more than 1.4 folds in tumor tissue of LLC-bearing mice after treatment with RNase A were assigned to metabolic and cancer-related pathways based on KEGG annotation and analysis by Gene Card.

We found that, in tumor tissue, RNase A treatment alters the expression of genes involved in metabolic pathways (25.1%), cancer-related events (responsible for cell growth, modulation of the cancer microenvironment and extracellular matrix components [17.9%]), pathways responsible for cell growth promoting and transforming activity (25.4%), transcription regulation (23.5%), tumor inductors and suppressors (6.2%), and miRNA-processing machinery (1.6%) (Table 1 and Supplementary Tables 1 –4).

### Metabolic cascades and pathways

The most significant alterations in gene expression were observed for the following metabolic pathways: amino sugar and nucleotide sugar metabolism; amino acids and nucleotide metabolism (four upregulated and eight downregulated genes, Table 1); carbohydrate metabolism (nine upregulated and eight downregulated genes, Table 1); metabolism of lipids and fatty acids (eleven upregulated and seven downregulated genes, Table 1). Oxidative phosphorylation (OXPHOS, six upregulated and three downregulated genes, Table 1, Supplementary Figure 1) and inositol phosphate metabolism (five upregulated and one downregulated genes, Table 1) were among those metabolic cascades that also undergo changes after RNase A treatment. Among key components of inositol phosphate metabolism, we observed upregulation for inositol(myo)-1(or 4)-monophosphatase 1 (*Impa 1*), an enzyme that dephosphorylates myo-inositol monophosphate to generate free myo-inositol, a precursor of phosphatidylinositol, and thus an important modulator of intracellular signal transduction (Supplementary Table 1).

We also detected changes in cytochrome P450-associated metabolism (one upregulated and three downregulated genes, Table 1), nicotinate and nicotinamide metabolism (two upregulated and one downregulated genes, Table 1), and glutathione metabolism (two upregulated and three downregulated genes, Table 1).

### Cancer-related events

Among cancer-related events (related to cancer progression, dissemination, and vitality), we observed changes in the expression of the genes involved in angiogenesis (two upregulated and four downregulated genes, Table 1), apoptosis (eight upregulated and twelve downregulated genes, Table 1), and cell cycle control and transformation (four upregulated and five downregulated genes, Table 1). The most affected events were cell adhesion, migration, invasion, and apoptosis (eight upregulated and twelve downregulated genes, Table 1). Among the eight upregulated genes observed for cell adhesion, migration, and invasion, we detected three genes positively regulating cell adhesion and affecting tumor dissemination (*Ptpn14*, *Myl12b*, and *Emp2*) (Table 1, Supplementary Table 1). Among the downregulated genes, we detected *Smoc2*, which promotes proliferation and migration.

Among the upregulated genes in apoptosis, we detected five genes encoded proteins that function as positive apoptosis inductors, both for caspase-dependent and mitochondrial pathways (*Pcpb4*, *Faim*, *Pycard*, *Plekhf1*, and *Dapk1*). Among the downregulated genes involved in apoptosis, we detected two genes encoding negative apoptosis regulators (*Hipk3* and *Bcl2l2*).

### Cancer-related signaling pathways

Of the cancer-related signaling pathways, we detected a negative regulation of some tumor-promoting pathways in tumor tissue after RNase A treatment, including the PI3K/AKT (three upregulated and twelve downregulated genes), TGF-β (two upregulated and six downregulated genes), JAK/STAT (two upregulated and eleven downregulated genes), and canonical WNT signaling (six upregulated and eleven downregulated genes) pathways (Supplementary Tables 1 and 2, Supplementary Figures 2, 4, and 5). The strong negative regulation of the TGF-β signaling pathway was associated with an upregulation of *Fam89b*, which is a TGF-β pathway suppressor (Table 1, Supplementary Figure 5 and Supplementary Tables 1 and 2). Some changes were also detected for the MAPK pathway (six upregulated and eleven downregulated genes, Supplementary Figure 3) and RAS and calcium signaling pathways (Table 1, Supplementary Tables 1 and 2).

### Cancer-associated genes and tumor suppressors

We also detected changes in the expression of genes usually considered as cancer-associated and whose increased expression is typical for various tumor types. In LLC tumors after RNase A treatment, we detected a downregulation of twelve such genes (Table 1, Supplementary Tables 1 and 2). Tumor suppressors were also affected by RNase A treatment (the upregulation of three genes and downregulation of four genes).

### Genes associated with miRNA biogenesis

We have previously reported that the antitumor and antimetastatic activities of RNase A are associated with changes in the miRNA profiles of blood serum and tumor tissue [20]. Here, we found that five genes were downregulated, including *Lin28a*, *Zcchc6*, and *Zcchc11*, which act as suppressors during the biogenesis of the let-7 miRNA family and in the terminal processing of miRNA precursors (Table 1, Supplementary Table 2).

### Genes involved in transcription regulation

Interestingly, transcription was greatly affected by RNase A. We found that RNase A treatment resulted in significant changes in the expression of genes encoding components of the transcription machinery in tumor tissue (Supplementary Tables 3 and 4). Transcriptional regulators (five upregulated and 24 downregulated genes) and transcription factors (16 upregulated and 13 downregulated genes) were mostly affected. Transcriptional activators and co-activators (three upregulated and three downregulated genes) and repressors and corepressors (five upregulated and four downregulated genes) were also affected (Supplementary Tables 3 and 4).

### Validation of expression level of genes - key members of pathways affected by RNase A

To validate our sequencing data, we tested the expression of some genes in tumor tissue after the treatment with RNase A by quantitative real-time PCR (qRT-PCR). At this stage we chose both up- and downregulated genes belonging to different processes and pathways promoting tumor survival and progression. Genes characterized by the highest LogFold but had low abundance in L_C_ (close to 1) were not consider for validation. As a result following genes were chosen: *Mtap* (metabolism of amino sugars, nucleotide sugars, amino acids and nucleotides), *Angptl4* (angiogenesis), *Fam89b* (a negative regulator of TGF-β signaling), *Serpinf1* (Wnt signaling pathway), and *Dusp6* and *Map2k4* (MAPK signaling pathway). Genes *Mtap* (LogFold 0.71), *Angptl4* (LogFold 0.62), and *Serpinf1* (LogFold 0.94) were chosen due to best ratio value in L_C_ (RPKM)/LogFold (L_R_/L_C_) in their pathways. *Fam89b* and genes belonging to MAPK pathways were chosen on the base of its significance for pathway functioning.

**Figure 4:**
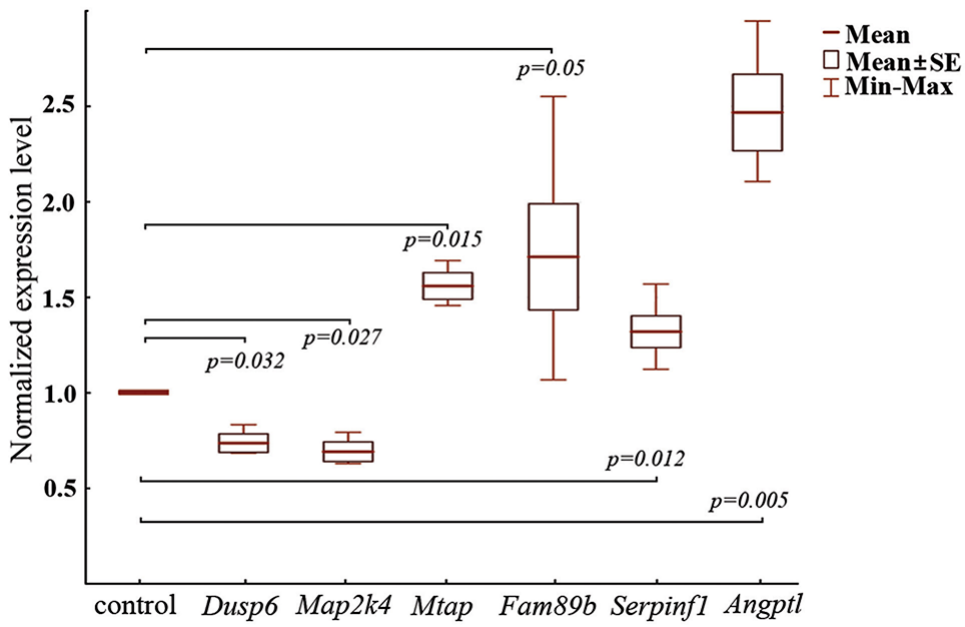
RT-qPCR analysis of expression levels of mRNA of *Dusp6*, *Fam89b*, *Map2k4*, *Mtap*, *Serpinf1*, and *Angptl4* genes in tumor tissue of LLC-bearing mice after treatment with RNase A The expression of mRNAs was normalized to *Hprt1* and *Ywhaz*. Data are presented as mean ± SE. The level of the corresponding gene in the control (mice with LLC treated with saline buffer) was set at 1.

In this experiment, we used rRNA-depleted samples of total RNA isolated from tumor tissue of RNase A treated and control mice. We found that *Dusp6* (1.3-fold) and *Map2k4* (1.5-fold) were downregulated following RNase A treatment (Figure 4, Table 2), which is in line with our sequencing data (Table 2). *Mtap* (1.6-fold), *Fam89b* (1.7-fold), *Serpinf1* (1.3-fold), and *Angptl4* (2.5-fold) (Figure 4) were upregulated following RNase A treatment, that also correlate with our sequencing data (Table 2).

**Table 2:** Comparison of levels of gene expression validated by qRT-PCR and sequencing

Gene	qRT-PCR data	Sequencing data
*Mtap*	↑1.6	↑1.6
*Angptl4*	↑2.5	↑1.5
*Fam89b*	↑1.7	↑1.6
*Serpinf1*	↑1.3	↑1.9
*Dusp6*	↓1.3	↓1.5
*Map2k4*	↓1.5	↓1.6

## DISCUSSION

Ribonucleases (RNases) are potentially cytotoxic by virtue of their ability to degrade RNA and, therefore, to inhibit protein biosynthesis at transcription and translation stages. The cytotoxic effects of RNase are associated with: (1) catalytic cleavage of available RNA, including tRNA, rRNA, mRNA, and non-coding RNA (microRNAs), that affects gene expression [18, 21-24]; (2) non-catalytic electrostatic interaction of the exogenous enzyme with cell components [25]; (3) modulation of the membrane calcium-dependent potassium channels and ras-oncogene functions [16, 26, 27]; (4) induction of apoptosis [28, 29]; and (5) the regulation of interplaying pathways [25, 30, 31].

Recently, we have shown that the RNase A-mediated inhibition of tumor growth and metastasis spreading in a murine LLC model [11-13] is accompanied by changes in the tumor tissue and blood miRNA profiles [20]. Next, we performed whole transcriptome analysis of tumor tissue after RNase A treatment and detected 966 differentially expressed transcripts involved in metabolic and signal transduction pathways important for regulation of cancer cell proliferation, survival, and maintenance of malignancy (Table 1, Supplementary Tables 1 –4). We also detected some changes in the activity of transcription machinery consisted in change of the spectrum of transcriptional activators, repressors, regulators and transcription factors (Supplementary Tables 3 and 4).

Among strongly affected metabolic pathways, we detected amino sugar and nucleotide sugar metabolism, amino acids and nucleotide metabolism, carbohydrate metabolism, and metabolism of lipids and fatty acids. OXPHOS (Supplementary Figure 1) and inositol phosphate metabolism were also significantly upregulated.

During the last five years, considerable knowledge has been accumulated on the bioenergetics of cancer cells, leading to a better understanding of the regulation of energy metabolism during oncogenesis. In tumor cells, in order to adapt the mechanisms of energy production to microenvironmental changes, as well as to differences in tumor energy needs or biosynthetic activity, there is interplay between glycolysis and OXPHOS. This metabolic flexibility is used to survive under hypoxia. Some studies have reported a reduction in OXPHOS capacity in multiple cancer types, whereas others have reported the opposite [32]. Here, we observed an increase in OXPHOS processes following RNase A treatment (Table 1, Supplementary Figure 1 and Supplementary Tables 1 and 2). Many tumors types are characterized by an absence of respiration, despite the presence of high oxygen concentrations (known as the Warburg effect) [33]. This is because of impaired mitochondrial function [34], resulting in an inhibition of OXPHOS processes. Thus, increased OXPHOS activity could be considered as evidence of a reversal of the cancerous phenotype. NADPH biogenesis, which is an essential mechanism by which both normal and cancerous cells maintain redox balance [35], was shown to be slightly upregulated after RNase A exposure. Given all aforementioned, we can conclude that these data are evidence for the increased energy demands of tumor cells following RNase treatment. Obtained data are in line with data about other ribonucleases, with the example of a nuclear-directed pancreatic ribonuclease PE5, which was shown to downregulate of multiple genes in cancer cells involved in deregulated metabolic pathways [36].

RNase A treatment resulted in re-arrangement of functioning of some pathways including angiogenesis, apoptosis, cell cycle control, and cell-cell contacts that lead eventually to positive regulation of cell adhesion and inhibition of tumor dissemination. Positive event in cell adhesion is an increase in expression of *Ptpn14* that plays a role in the regulation of cell-cell adhesion, cell-matrix adhesion, cell migration, cell growth, and also regulates TGF-β gene expression, thereby modulating epithelial-mesenchymal transition [37, 38]. Ptpn14 also functions as a tumor suppressor [39]. An increase in *Emp2* expression, which regulates the plasma membrane expression of the integrin heterodimers Itga6-Itgb1, Itga5-Itgb3, and Itga5-Itgb1, thereby resulting in modulation of cell-matrix adhesion [40], and *Myl12b*, which participates in focal adhesion and at tight junctions [41], should be considered as positive events in cell adhesion. An important finding of our study is the decrease in expression of *Smoc2* that stimulates endothelial cell proliferation, migration, as well as angiogenesis [42].

Some ribonucleases were found to participate in the control of cell proliferation and migration. Mutations in RNase L gene might promote enhanced cell migration and metastasis while RNase L knockdown in PC3 cells increased tumor growth and metastasis *in vivo* [43, 44]. Human secreted ribonuclease RNASET2 operates through the control of both the cytoskeletal actin assembly [45-47] and cell motility/migration patterns [48].

Changes in the expression of genes involved in apoptosis allow concluding about the activation of intrinsic mitochondrial pathway in tumor tissue after exposure to RNase A. We detected an upregulation of the genes *Pycard*, *Plekhf1*, and *Dapk1*, which activate the mitochondrial apoptotic pathway. The increase in *Faim* expression, which encodes a protein that participates in protection against death receptor-triggered apoptosis [49], also indicates an enhancement of the intrinsic mitochondrial pathway. The decrease in the expression of *Gzmb* encoding protein which activates caspase-3, -7, -9, and 10 and promotes caspase-dependent apoptosis, is further evidence for the attenuation of this pathway functioning. The upregulation of *Pcbp4* expression, which affects cell proliferation by inducing apoptosis and cell cycle arrest in the G(2)-M phase and is implicated in lung tumor suppression [50], as well as the downregulation of negative apoptosis regulators, are the key changes in the regulation of apoptosis caused by RNase A.

We detected a negative regulation by RNase A of multiple tumor-promoting pathways, including the PI3K/AKT, TGF-β, JAK/STAT and canonical WNT signaling pathways (Supplementary Figure 2, 4 and 5 and Supplementary Tables 1 and 2). The MAPK and PI3K/AKT pathways govern fundamental physiological processes, such as cell proliferation, differentiation, metabolism, cytoskeleton reorganization, and cell death and survival. Constitutive activation of these signal transduction pathways is a hallmark of cancer and dysregulation, and these pathways have been implicated in the initiation, progression and metastatic spread of lung cancer [51]. The JAK/STAT pathway is a key player in the development of various tumor types (e.g., gastric cancer [52], pancreatic cancer [53], and hematological malignancies [54]). Among the genes involved in the JAK/STAT and PI3K/AKT pathways, we detected a downregulation of the genes encoding the Januse kinases Jak2 and Jak3, which participate in the activation of proteins of STAT family, thereby promoting tumorigenesis [55].

Based on our findings, we cannot determine whether the MAPK pathway is likely to be overall upregulated or downregulated by RNase A treatment (Supplementary Figure 3 and Supplementary Tables 1 and 2). We detected an upregulation of *Rras*, which displays tumor-promoting activity, and a downregulation of *Dusp6*, which participates in the inactivation of MAP kinases (Table 1). Nevertheless some positive effect on expression of the genes of MAPK pathway took place. We also detected a downregulation of *Trib3*, which regulates the activation of MAP kinases [56] and blocks Akt kinases promoting cell proliferation and survival [57]. We also observed downregulation of *Map2k4*, which has been shown to be overexpressed in osteosarcomas and associated with a poor response to treatment, tumor progression, and worse overall survival [58] (Table 1). Thus, RNase A alters the MAPK pathway by some extent, but no conclusions can be done on its overall up- or downregulation.

TGF-β-induced signaling pathways have either tumor-suppression or tumor-promoting effects in a cancer type-specific and stage-dependent manner [59]. At a later stage of tumor progression, TGF-β exerts metastasis-promoting activity associated with epithelial-to-mesenchymal transition, modulation of cancer microenvironment and extracellular matrix components, inflammation, and immune suppression [60]. Here, we detected a strong negative regulation of the TGF-β signaling pathway in tumor tissue after RNase A treatment, which was associated with the upregulation of *Fam89b* which is a TGF-β pathway suppressor (according to UniProt data, by sequence similarity).

Abnormal activation of Wnt signaling has been implicated in many cancer types, including gastrointestinal cancers, leukemia, melanoma, and breast cancer [61]. Here, we observed a downregulation of *Lrrfip2* following RNase A treatment which is an activator of the canonical Wnt signaling pathway [62], and together with *Shisa2* and *Hmgxb4* promotes the attenuation of Wnt signaling (from UniProt, by similarity). Thus, we conclude that RNase A negatively regulates the canonical Wnt pathway in LLC.

**Figure 5:**
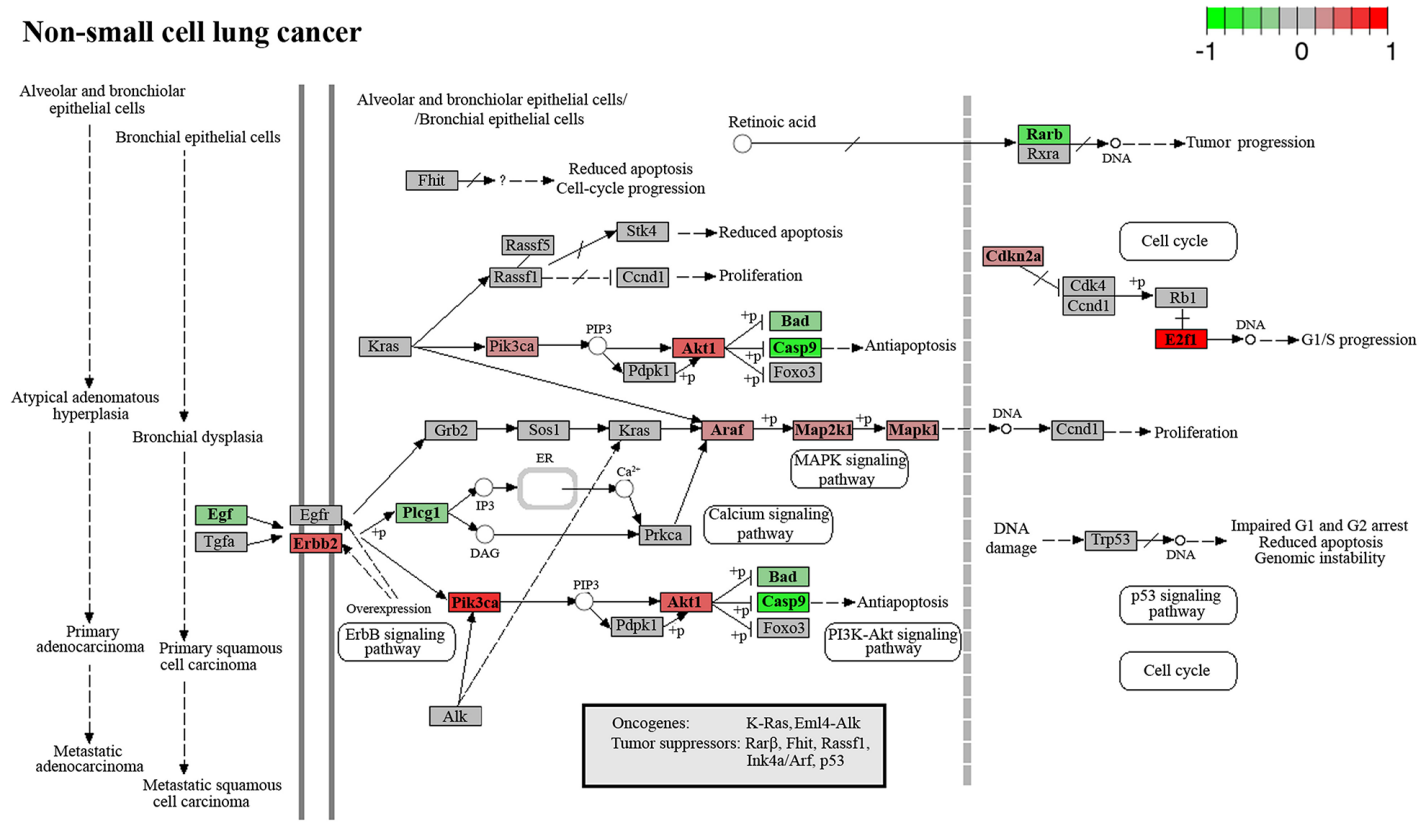
Genes involved in the progression of non-small cell lung cancer

LLC has an epithelial origin and is related to human non-small cell lung cancer (NSCLC) [63]. Therefore, we attempted to evaluate the interplay between cancer-related signaling pathways affected by RNase A and NSCLC development pathways. According to our sequencing data, the PI3K/AKT, MAPK, and calcium signaling pathways, which are directly involved in NSCLC progression, are downregulated by RNase A treatment (Figure 5). Downregulation of these particular pathways can give an impact in observed antitumor effect of RNase A manifested in retardation of primary tumor growth and inhibition of metastasizing treatment [11-13].

Another important result of our study is the downregulation of genes encoding suppressors of miRNA biogenesis (*Lin28a*, *Zcchc6*, and *Zcchc11*) (Table 1 and Supplementary Table 2). Lin28a prevents terminal processing of the let-7 family of miRNA [64]. Zcchc6 and Zcchc11 act as suppressors of miRNA biogenesis by mediating the terminal uridylation of some miRNA precursors, including pre-let-7 (by Zcchc6 and Zcchc11) and mir-107, mir-143, and mir-200c (by Zcchc11) [65]. These data might account for the observed upregulation of a wide spectrum of miRNAs (including miRNAs of the let-7 family) in tumor tissue after treatment with RNase A [20].

Our findings are in line with other studies of the effect of cytotoxic ribonucleases on gene expression profile of tumor cells. It was shown that onconase significantly affect apoptosis, transcription, inflammation and the immune response in human mesothelioma cells [66]. Pathways affected by onconase include MAPK signaling, cytokine-cytokine-receptor interactions, and Jak-STAT signaling [66]. In ovarian cancer cells onconase strongly up-regulated genes involved in transcription regulation, cell cycle and apoptosis, immune and stress response [67] while nuclear-directed human pancreatic RNase PE5 down-regulated multiple genes coded for proteins engaged in cell adhesion/migration and enzymes involved in deregulated metabolic pathways [68].

**Figure 6:**
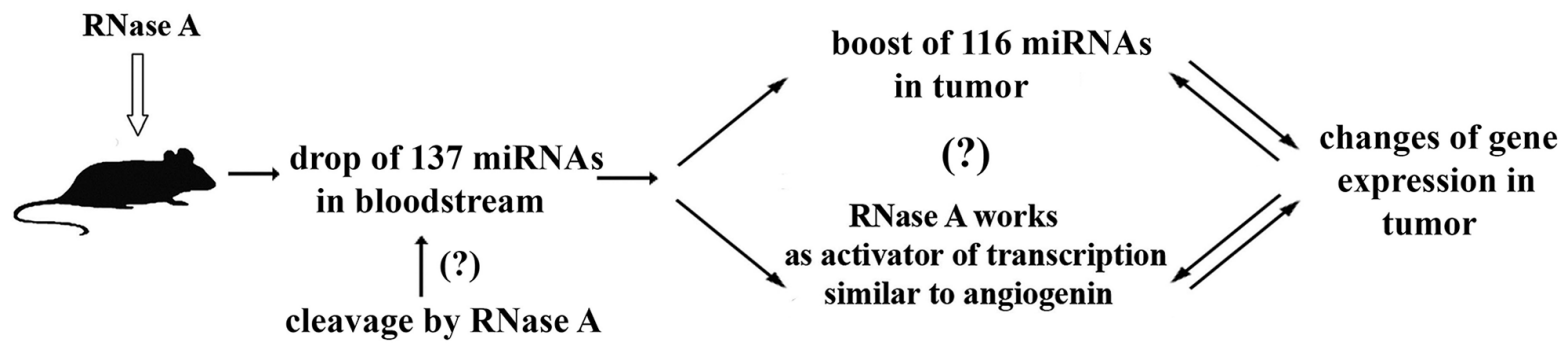
Proposed mechanism of antitumor activity of RNase A RNase A therapy resulted in the boost of 116 miRNAs in tumour tissue and drop of 137 miRNAs in the bloodstream of mice with intramuscularly transplanted LLC and in the changes in the expression of 966 transcripts in tumor cells.

A possible mechanism of the antitumor activity of RNase A is depicted in Figure 6. We propose that, as soon as RNase A reaches the bloodstream after i.m. injection, its main targets are extracellular RNAs, including a whole set of non-coding RNAs (and miRNAs). In line with this hypothesis we observed previously a significant drop in miRNA levels in the bloodstream after RNase A exposure but no evidences of direct miRNA cleavage by RNase A in the blood have been obtained. Since miRNA/Ago2 complexes as well as miRNA in the microvesicles are known to participate in the distant (similar to endocrine) regulation we observed global changes in miRNA profiling and whole transcriptome of tumor tissue. Here, we report changes in the expression of 966 transcripts following RNase A treatment. Despite reports of the neutralization of RNase A by cytosolic RNase inhibitor (RI) [69], we cannot exclude a potential effect of RNase A on intracellular RNAs because we have no data on intracellular localization of RI and the ability of extracellular RNase A to penetrate into the cells. Similar to angiogenin, RNase A can function as a transcriptional activator, as discussed in an earlier publication [20].

Our data suggest that the antitumor activity and the decrease in invasion potential of tumor cells by RNase A can be accounted for enhanced energy cascade functioning, rearrangement of cancer-related events regulating cell growth and dissemination, and attenuation of signaling pathways having tumor-promoting activity. Thus, we conclude that the antitumor effect of RNase A is realized via multilevel regulation that induces a systemic antitumor response.

## MATERIALS AND METHODS

### Tumor transplantation and design of animal experiment

All animal procedures were carried out in strict accordance with the recommendations for proper use and care of laboratory animals (ECC Directive 2010/63/EU). The protocol and this study were specifically approved by the Committee on the Ethics of Animal Experiments of the Administration of the Siberian Branch of the Russian Academy of Sciences (permit number: 5-06-2015).

Lewis lung carcinoma (LLC) cells having epithelial origin [63] were generously provided by Dr N.A. Popova (Institute of Cytology and Genetics, SB RAS).

At the start of the experiments, animal weight (mean ± SD) was 20.2 ± 1.5 g. To perform LLC studies, ten animals per group were used. The number of animals in each group was chosen in accordance with the requirement to provide statistically significant data.

Twelve- to 14-week-old female C57Bl/6J mice were used. LLC cells (10^6^) in 0.1 ml of saline buffer were injected (i.m.) into the right thighs of mice and, on the 4^th^ day after tumor transplantation, mice were treated with saline buffer (n=10) or RNase A (Sigma, USA) at a dose of 0.7 μg/kg (n=10). RNase A was administered in a volume of 0.1 ml i.m. daily, except for weekends. The total number of injections was 10. On day 15 after tumor transplantation, tumor samples were collected 60 min after the last injection of RNase A.

### Sample processing and RNA extraction

Tumor pieces from mice treated with saline buffer or RNase A were pooled according to groups and homogenized. Total RNA was extracted immediately using TRIzol Reagent (Invitrogen, USA). The long RNA (>200 nt) fraction was separated from the short RNA (<200 nt) fraction using the mirVana miRNA Isolation Kit (Ambion, USA), according to the manufacturer’s protocols. Ribosomal RNAs were removed from the long RNA fraction using the RiboMinus Eukaryote Kit (Life Technologies, USA). Two rRNA-depleted fractions of total RNA were obtained: the RNA fraction from tumor tissue of control mice treated with saline buffer (RNA_C(-r)_) and an RNA fraction isolated from tumor tissue of mice treated with RNase A (RNA_R(-r)_).

### Preparation of DNA libraries and sequencing

DNA libraries were prepared from RNA using the SOLiD™ Whole Transcriptome Kit (Applied Biosystems, USA), in accordance with the manufacturer’s recommendations. In brief, rRNA-depleted fractions of total RNA (350 ng of each) were fragmented with RNase III and precipitated by ethanol in the presence of glycogen. The obtained RNA fragments (70 ng for each library) were hybridized with the adapter mix A and incubated for ligation for 16 h at 4°C, followed by reverse transcription (RT) and RNase H treatment. Large-scale PCRs were performed using 18 cycles, both for samples isolated from tumors of control and experimental groups. The cycling conditions were 95°C for 30 s, 62°C for 30 s, and 72°C for 30 s. The obtained adaptor-ligated PCR products were then cleaned using the QIAquik Qiagen Gel Extraction Kit (Qiagen, USA) and size-selected using 6% PAGE gel. The piece of the gel corresponding to 150–200 bp was cut-out, divided into four vertical pieces, and the two central pieces were used for the generation of cDNA by PCR (the same cycling conditions as above, 15 cycles). Desalting of the eluted PCR products was performed using the QIAquick Gel Extraction Kit (Qiagen, Germany). Then, the PCR products were quantified using the Quant-iT™ dsDNA HS Assay Kit (Invitrogen). Templated bead preparation, emulsion PCR, and deposition were performed and, as a result, two cDNA-libraries were constructed: L_C_ (saline treated) and L_R_ (RNase A treated). Libraries were sequenced using the standard SOLiD™ V5.5 (Applied Biosystems) protocols.

Libraries were run on a single slide, each on its quadrant, with 50 nucleotides read length on the SOLiD™ 5.5 system resulting, accordingly, in 5.3 × 10^7^ and 3.1 × 10^7^ total number of reads. Sequencing data have been submitted to the GEO Database (accession number GEO GSE63758).

### qPCR

The expression of *Dusp6*, *Fam89b*, *Map2k4*, *Mtap*, *Serpinf1*, and *Angptl4* were evaluated using qPCR. cDNA synthesis was performed in a total volume of 40 μl containing 5 μg of RNA_C(-r)_ or RNA_R(-r)_, RT buffer (50 mM Tris-HCl, pH 8.3, 75 mM KCl, 3 mM MgCl2), 10 mM DTT, 0.5 mM dNTPs, 100 pmol of random hexa-primers, and 20 units of M-MLV reverse transcriptase (ICBFM SB RAS, Russia). cDNA synthesis was carried out at 37°C for 60 min. PCR amplification was carried out in a total volume of 20 μl using 5 μl of cDNA (10^-2^ dilution), 0.6 units of Maxima Hot Start Taq DNA Polymerase (Thermo Scientific, USA), 1×PCR buffer, 1.5 mM MgCl_2_, 0.2 mM dNTPs, 1×EvaGreen (Biotium, USA), and 0.25 μM sense and antisense primers. The following primers were used: *Dusp6* sense 5′-CCTGGAAGGTGGCTTCAGTA-3′, *Dusp6* antisense 5′-AGTCCGTTGCACTATTGGGG-3′; *Fam89b* sense 5′-CAAGGAGATGGTGGGGCTG-3′, *Fam89b* antisense 5′-CTCCTCGTCATCAGACAGGC-3′; *Map2k4* sense 5′-CATGCAGGGTAAGCGCAAAG-3′; *Map2k4* antisense 5′- ATCCCAGTGTTGTTCAGGGG-3′; *Mtap* sense 5′-ATACTCCATTCGGCAAGCCAT-3′, *Mtap* antisense 5′-CTCTCTCAAGGACCCGCAAG-3′; *Serpinf1* sense 5′- TTCAAGGGGCAGTGGGTAAC-3′, *Serpinf1* antisense 5′- CAGGGGCAGGAAGAAGATGA-3′; *Hprt1* sense 5′-TTGCTGACCTGCTGGATTAC-3′, *Hprt1* antisense 5′- AGTTGAGAGATCATCTCCAC-3′; *Ywhaz* sense 5′-CCTCAACTTCTCTGTGTTCTA-3′, *Ywhaz* antisense 5′-TGCTTCATCTCCTTGGGTATC-3′. The reaction was performed with an initial preheating at 94°C for 4 min and 40 cycles of 94°C for 40 s, 60°C for 30 s, 72°C for 30 s, followed by a melting point determination. The expression level of each gene is indicated by the number of cycles needed for the cDNA amplification to reach a threshold. The amount of DNA is calculated from the number of cycles by using standard curves, and the results are normalized to *Hprt1* and *Ywhaz*. Relative gene expression was calculated using the standard BioRad IQ5 software (BioRad, USA). qPCR data were statistically processed using one-way ANOVA with post hoc Tukey test; p <0.05 was considered to be statistically significant. The statistical package STATISTICA version 10.0 was used for analysis.

### Gene mapping and annotation

Reads were mapped to the reference *Mus musculus* genome (version NCBI37) using Bioscope software v.1.3 with default mapping parameters. Transcript counts extraction and transformation to RPKMs (reads per kilobase per million reads), followed by analysis of differential expression, was performed with Cufflinks software v.2.0.1 (Center for Bioinformatics and Computational Biology, USA) [70]. Feature extraction, reads per kilobase per million reads in the library (RPKM), where RPKM = the number of reads of a specific mRNA/(size of mRNA[kb] × total number of reads in the library[mln]).

Genes were annotated to GO terms using the PANTHER Classification System (http://pantherdb.org). The comparative pathway mapping was done with the Pathview package for R statistical language. Before pathway mapping, Log2fold RPKM values were scaled to range between -1 and 1. Kyoto Encyclopedia of Genes and Genomes (KEGG) pathways were assigned to the assembled sequences using the online KEGG Automatic Annotation Server (http://www.genome.jp/kegg/kaas/). The Gene Card database (http://www.genecards.org) was used for functional gene analysis. The Mouse Genome Informatics was used for mouse gene symbols identification. Tool for transcriptome annotation based gene list functional enrichment analysis ToppFun (https://toppgene.cchmc.org) was used for pathway p-value.

## SUPPLEMENTARY MATERIALS FIGURES AND TABLES










