# Supplementary material for: The systemic tumor response to RNase A treatment affects the expression of genes involved in maintaining cell malignancy
Source: Oncotarget. 2017 Aug 12;8(45):78796–810. doi: 10.18632/oncotarget.20228 (PMC5667999; doi:10.18632/oncotarget.20228)
Supplement: Supplementary file 1 [file oncotarget-08-78796-s001.pdf]

The systemic tumor response to RNase A treatment affects the expression of genes involved in maintaining cell malignancy

SUPPLEMENTARY MATERIALS

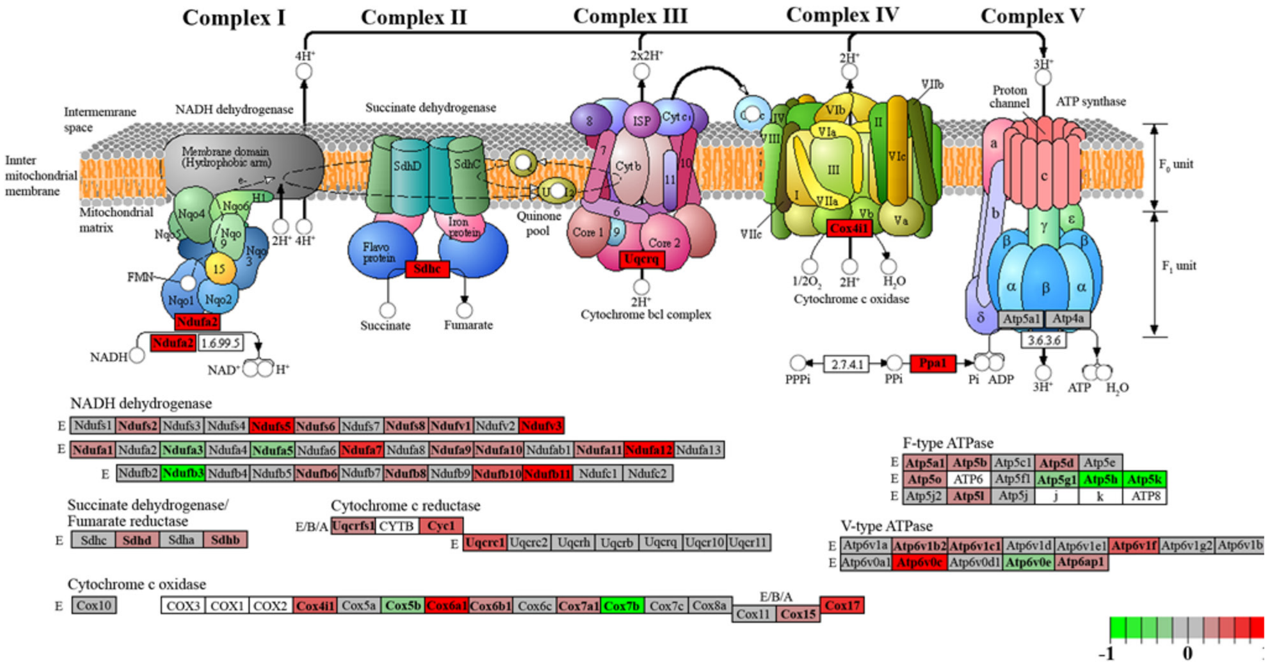

Supplementary Figure 1: Genes and components involved in oxidative phosphorylation.

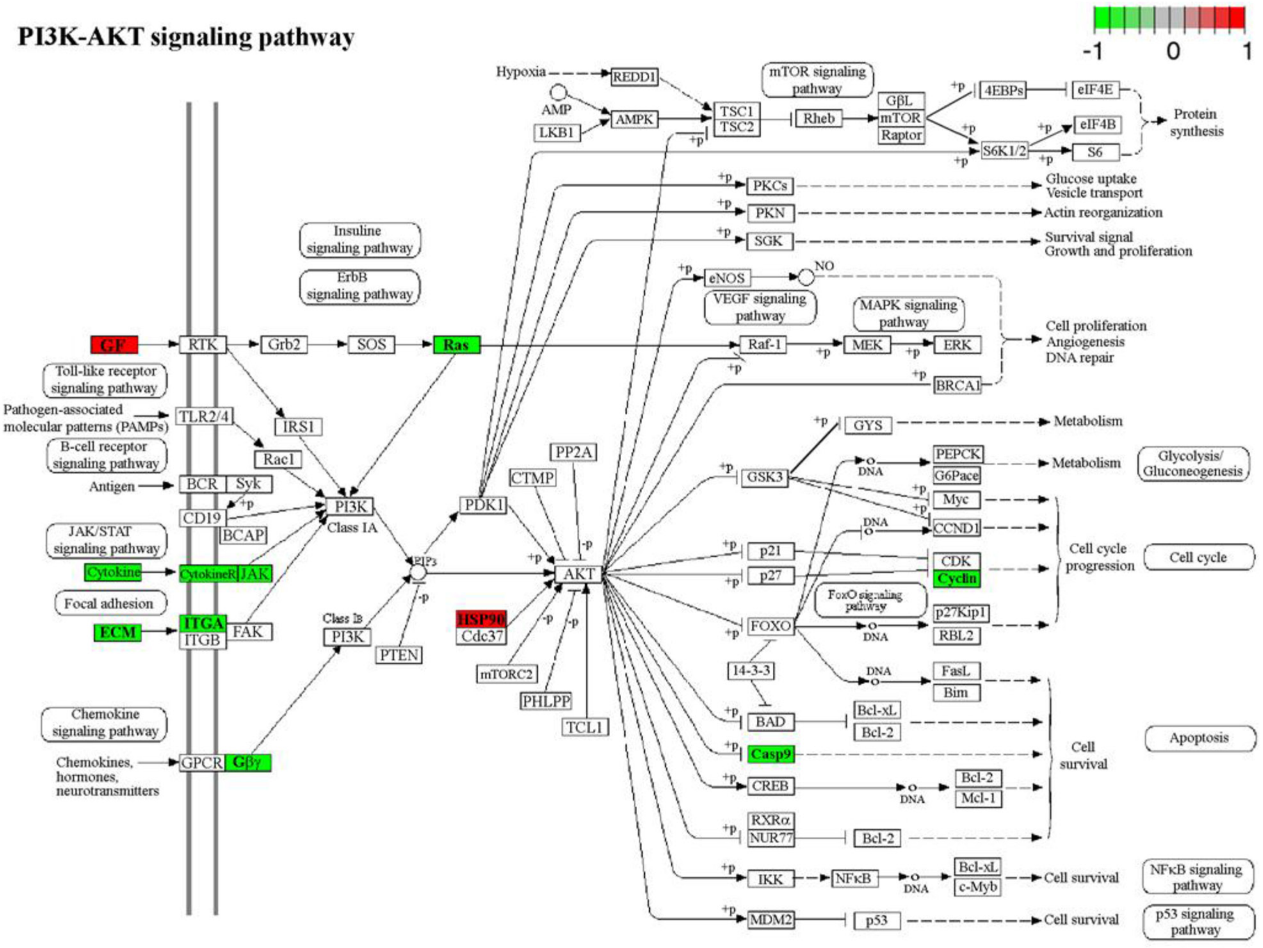

Supplementary Figure 2: Genes involved in the PI3K-AKT signaling pathway.

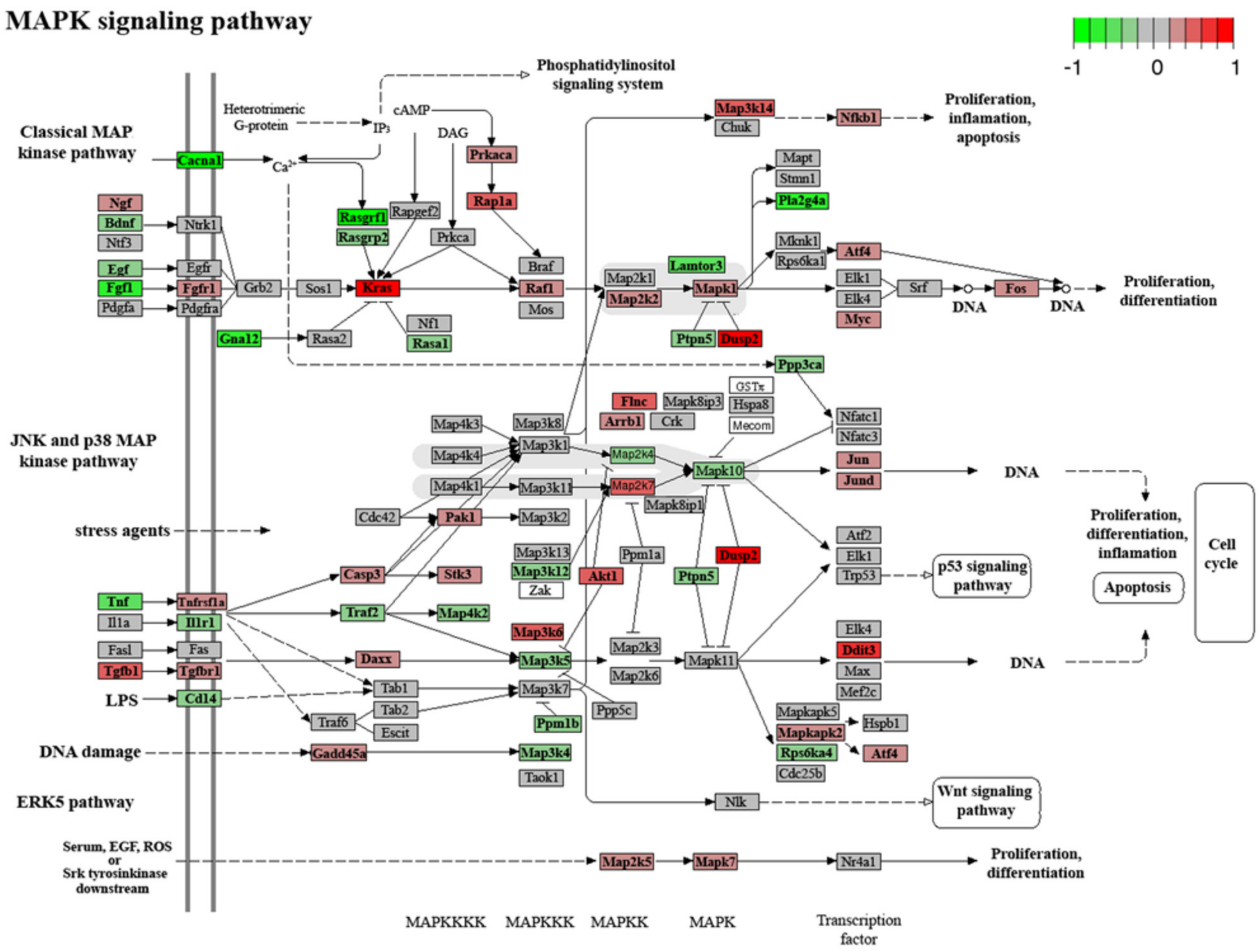

Supplementary Figure 3: Genes involved in the MAPK signaling pathway.

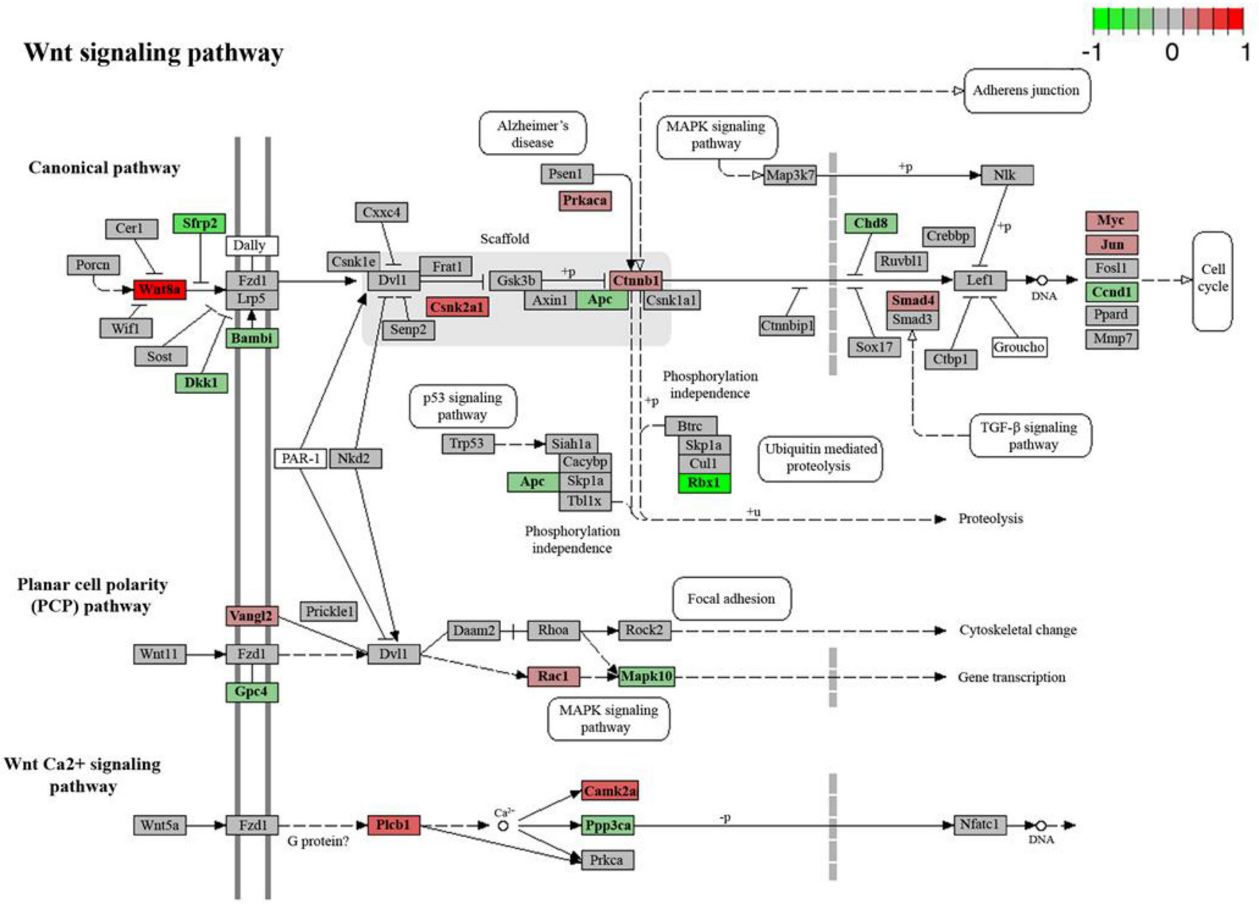

Supplementary Figure 4: Genes involved in the Wnt signaling pathway.

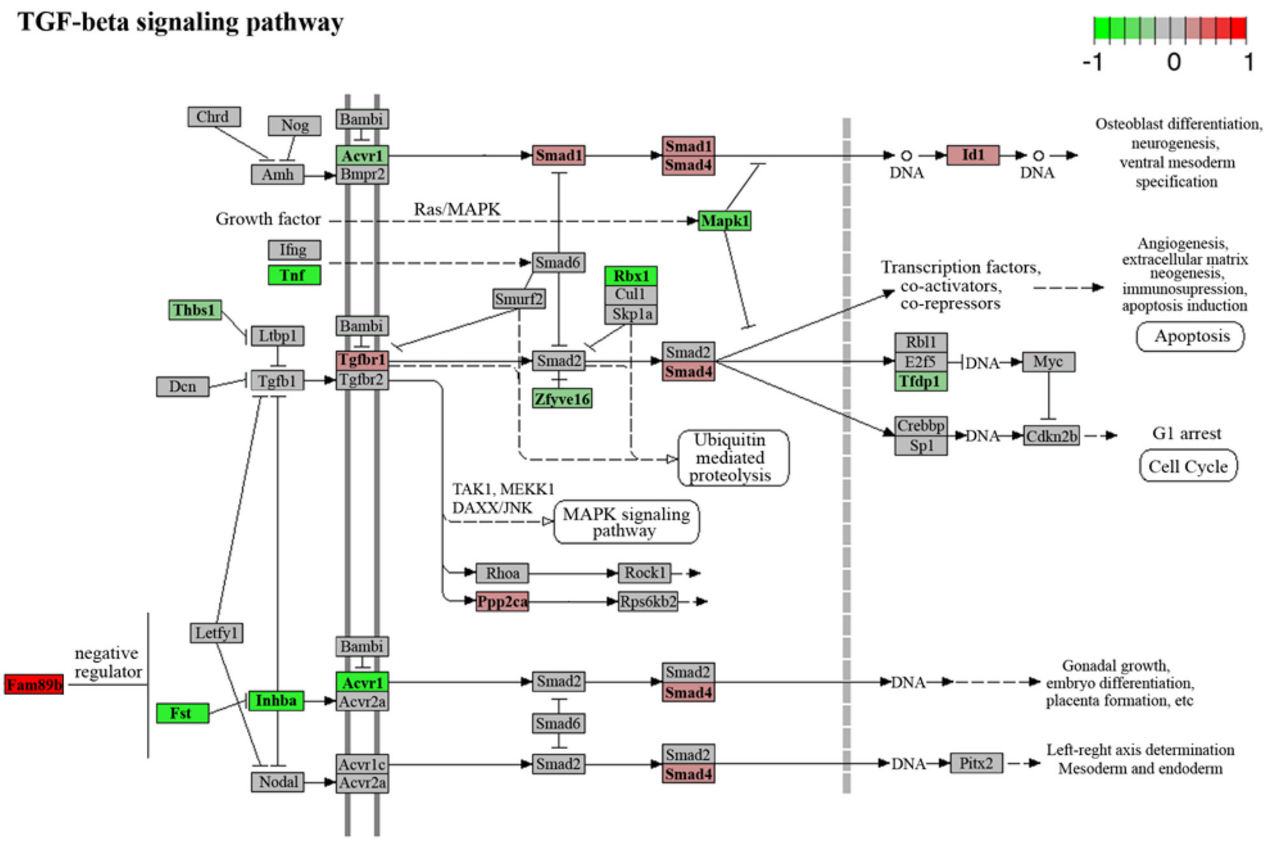

Supplementary Figure 5: Genes involved in the TGF-β signaling pathway.

Supplementary Table 1: Up-regulated genes in tumor of mice with LLC after RNase A treatment (L_C)

See Supplementary File 1

Supplementary Table 2: Down-regulated genes in tumor of mice with LLC after RNase A treatment (sorted by Value L_C)

See Supplementary File 2

Supplementary Table 3: Upregulated transcription-related genes in tumor of mice with LLC after RNase A treatment (sorted by Value L_R)

See Supplementary File 3

Supplementary Table 4: Down-regulated transcription-related genes in tumor of mice with LLC after RNase A treatment (sorted by Value L_C)

See Supplementary File 4
