# Supplementary material for: The systemic tumor response to RNase A treatment affects the expression of genes involved in maintaining cell malignancy
Source: Oncotarget. 2017 Aug 12;8(45):78796–810. doi: 10.18632/oncotarget.20228 (PMC5667999; doi:10.18632/oncotarget.20228)
Supplement: Supplementary file 2 [file oncotarget-08-78796-s002.docx]

**Supplementary Table 1:** Up-regulated genes in tumor of mice with LLC after RNase A treatment (L_C_).

| **Symbol** | **Gene name** | **Value L_C_, RPKM** | **Value L_R_, RPKM** | **Log2 (L_R_/L_C_)** | **p-value** | **q-value** |
| --- | --- | --- | --- | --- | --- | --- |
| **Metabolism related** | | | | | | |
| **Amino sugar and nucleotide sugar metabolism, amino acids and nucleotide metabolism** | | | | | | |
| *Uap1l1* | UDP-N-Acetylglucosamine pyrophosphorylase 1 like 1 | 17.34 | 26.29 | 0.6 | 0.00187 | 0.02923 |
| *Mtap* | Methylthioadenosine phosphorylase | 13.66 | 22.33 | 0.71 | 9.81E-05 | 0.00481 |
| *Cant1* | Calcium activated nucleotidase 1 | 6.52 | 11.51 | 0.82 | 0.00256 | 0.03514 |
| *Impdh2* | IMP (Inosine 5'-monophosphate) dehydrogenase 2 | 1.02 | 2.74 | 1.43 | 0.00339 | 0.04178 |
| **Carbohydrate metabolism** | | | | | | |
| *Pgls* | 6-Phosphoglucono-lactonase | 18.58 | 33.22 | 0.84 | 0.00027 | 0.00901 |
| *Gaa* | Glucosidase, alpha | 15.80 | 22.90 | 0.54 | 0.00144 | 0.02547 |
| *Bpgm* | 2,3-Bisphosphoglycerate mutase | 9.06 | 14.14 | 0.64 | 0.00292 | 0.03832 |
| *Mcee* | Methylmalonyl CoA epimerase | 8.60 | 21.53 | 1.32 | 1.59E-05 | 0.00166 |
| *B4galt2* | UDP-Gal:BetaGlcNAc beta 1,4- galactosyltransferase, polypeptide 2 | 4.85 | 8.40 | 0.79 | 0.0037 | 0.04364 |
| *Xylb* | Xylulokinase | 2.32 | 4.99 | 1.10 | 1.92E-05 | 0.00188 |
| *Pdk4* | Pyruvate dehydrogenase kinase 4 | 1.34 | 2.72 | 1.02 | 0.00103 | 0.02062 |
| *Aldoc* | Aldolase, fructose-bisphosphate C | 0.64 | 2.39 | 1.90 | 0.00094 | 0.0197 |
| *Lalba* | Lactalbumin alpha | 0 | 3.10 | - | 0.00011 | 0.00517 |
| **Cytochrome P450-associated metabolism** | | | | | |  |
| *Akr7a5* | Succinic semialdehyde reductase | 5.48 | 9.43 | 0.78 | 0.00315 | 0.04015 |
| **Phosphatidylnositol** | | | | | | |
| *Impa1* | Inositol monophosphatase 1 | 15.79 | 22.55 | 0.51 | 0.0038 | 0.04436 |
| *Gpaa1* | Glycosylphosphatidylinositol anchor attachment 1 | 7.43 | 12.18 | 0.71 | 0.00175 | 0.02796 |
| *Ip6k2* | Inositol hexakisphosphate kinase 2 | 2.89 | 8.04 | 1.48 | 4.76E-07 | 0.00016 |
| *Plcd1* | Phospholipase C, delta 1 | 2.64 | 4.78 | 0.852 | 0.00236 | 0.03338 |
| *Pigb* | Phosphatidylinositol glycan anchor biosynthesis class B | 1.74 | 4.69 | 1.43 | 0.00128 | 0.0237 |
| **Metabolism of lipids and fatty acids** | | | | | | |
| *Ech1* | Enoyl CoA hydratase 1, peroxisomal | 37.98 | 54.91 | 0.53 | 0.00310 | 0.03980 |
| *Hadh* | Hydroxyacyl-CoA dehydrogenase | 11.77 | 25.37 | 1.11 | 6.51E-08 | 3.85E-05 |
| *Eci2* | Enoyl-CoA delta isomerase 2 | 8.82 | 19.01 | 1.11 | 0.00017 | 0.00663 |
| *Acadvl* | Acyl-CoA dehydrogenase, very long chain | 7.85 | 13.03 | 0.73 | 0.00103 | 0.02062 |
| *Pld3* | Phospholipase D family, member 3 | 6.50 | 11.38 | 0.81 | 0.00045 | 0.01241 |
| *Cbr4* | Carbonyl reductase 4 | 2.33 | 5.91 | 1.34 | 0.0011 | 0.02151 |
| *Acat2* | Acetyl-CoA acetyltransferase 2 | 1.86 | 4.88 | 1.39 | 2.52E-05 | 0.00215 |
| *Phospho1* | Phosphoethanolamine/ phosphocholine phosphatase | 1.24 | 3.77 | 1.60 | 0.00331 | 0.04151 |
| *Plcxd2* | Phosphatidylinositol specific phospholipase C X domain containing 2 | 1.20 | 2.16 | 0.84 | 0.00105 | 0.02091 |
| *Acsbg1* | Lipidosin | 0.68 | 1.75 | 1.37 | 0.00229 | 0.03303 |
| *St3gal5* | Ganglioside GM3 synthase | 0.26 | 1.40 | 2.44 | 0.00243 | 0.03388 |
| **Oxidative phosphorylation** | | | | | | |
| *Ndufv3* | NADH dehydrogenase (ubiquinone) flavoprotein 3, 10kDa | 17.75 | 42.63 | 1.26 | 5.19E-05 | 0.00319 |
| *Cox8a* | Cytochrome C oxidase subunit VIIIA (ubiquitous) | 24.20 | 41.03 | 0.76 | 0.00366 | 0.04343 |
| *Foxred1* | FAD-dependent oxidoreductase domain containing 1 | 10.74 | 16.27 | 0.6 | 0.00324 | 0.04087 |
| *Ndufb11* | NADH dehydrogenase (ubiquinone) 1 beta subcomplex, 11, 17.3kDa | 5.04 | 14.96 | 1.57 | 3.72E-05 | 0.00273 |
| *Bcs1l* | BC1 (Ubiquinol-cytochrome C reductase) synthesis-like | 3.33 | 6.82 | 1.04 | 0.00038 | 0.0114 |
| *Cox18* | COX18 Cytochrome C oxidase assembly factor | 2.06 | 6.37 | 1.63 | 5.05E-05 | 0.00317 |
| **Nicotinate and nicotinamide metabolism** | | | | | | |
| *Nmnat3* | Nicotinamide nucleotide adenylyltransferase 3 | 2.02 | 4.29 | 1.08 | 0.00073 | 0.01671 |
| *Art5* | ADP-ribosyltransferase 5 | 0.83 | 2.58 | 1.63 | 0.00147 | 0.02569 |
| **Glutathione metabolism** | | | | | | |
| *Chac1* | ChaC glutathione-specific gamma-glutamylcyclotransferase 1 | 4.66 | 8.87 | 0.93 | 0.00091 | 0.01927 |
| *Haghl* | Hydroxyacylglutathione hydrolase-like | 2.45 | 8.07 | 1.72 | 4.99E-05 | 0.00314 |
| **Cancer related** | | | | | | |
| **Angiogenesis** | | | | | | |
| *Angptl4* | Angiopoietin-Like 4 | 14.83 | 22.73 | 0.62 | 0.00148 | 0.02575 |
| *Adamts10* | ADAM metallopeptidase with thrombospondin type 1 motif 10 | 2.23 | 3.95 | 0.83 | 0.00166 | 0.0271 |
| *Robo4* | Roundabout guidance receptor 4 | 1.03 | 2.11 | 1.04 | 0.002 | 0.03073 |
| **Apoptosis** | | | | | | |
| *Pcbp4* | Poly(RC) binding protein 4 | 47.48 | 69.55 | 0.55 | 0.00089 | 0.01913 |
| *Steap3* | Tumor suppressor-activated pathway protein 6 | 16.05 | 23.19 | 0.53 | 0.00242 | 0.03388 |
| *Ctsh* | Cathepsin H | 13.46 | 20.99 | 0.64 | 0.00211 | 0.03174 |
| *Faim* | Fas apoptotic inhibitory molecule | 5.57 | 13.60 | 1.29 | 2.18E-05 | 0.00199 |
| *Pycard* | Caspase recruitment domain-containing protein 5 | 4.75 | 8.99 | 0.92 | 0.00024 | 0.00846 |
| *Plekhf1* | Lysosome-associated apoptosis-inducing protein containing PH and FYVE domains | 1.57 | 5.74 | 1.87 | 2.63E-06 | 0.00054 |
| *Nol3* | Nucleolar protein 3 (apoptosis repressor with CARD Domain) | 0.39 | 1.88 | 2.28 | 0.00011 | 0.00519 |
| *Dapk1* | Death associated protein kinase 1 | 0.49 | 1.56 | 1.66 | 7.83E-06 | 0.00103 |
| **Cell adhesion, migration, invasion** | | | | | | |
| *Rap2a* | RAP2A, member of RAS oncogene family | 7.50 | 10.91 | 0.54 | 0.00381 | 0.04438 |
| *Myl12b* | Myosin, light chain 12B, regulatory | 5.64 | 13.62 | 1.27 | 4.03E-05 | 0.00280 |
| *Emp2* | Epithelial membrane protein | 5.40 | 8.84 | 0.71 | 0.00081 | 0.01776 |
| *Ptpn14* | Protein tyrosine phosphatase, non-receptor type 14 | 3.85 | 5.70 | 0.56 | 0.00152 | 0.02591 |
| *Bcas3* | Microtubule associated cell migration factor | 1.83 | 3.52 | 0.94 | 0.00246 | 0.03418 |
| *Abi3* | ABI family member 3 | 1.05 | 3.52 | 1.74 | 8.34E-06 | 0.00103 |
| *Ajap1* | Adherens junctions associated protein 1 | 1.28 | 2.83 | 1.14 | 0.00054 | 0.01361 |
| *Jup* | Desmoplakin III | 0.82 | 1.78 | 1.11 | 0.00053 | 0.01352 |
| *Macrod1* | MACRO domain containing 1 | 0.72 | 2.79 | 1.96 | 0.0031 | 0.03979 |
| **Cell cycle control,** **transformation** | | | | | | |
| *Usp10* | Ubiquitin specific peptidase 10 | 15.57 | 22.53 | 0.53 | 0.00187 | 0.02923 |
| *Mad2l2* | MAD2 mitotic arrest deficient-like 2 (yeast) | 3.31 | 6.89 | 1.06 | 0.00206 | 0.03139 |
| *Cdc26* | Cell division cycle 26 | 1.14 | 5.82 | 2.35 | 0.00042 | 0.01186 |
| *Rnf122* | Ring finger protein 122 | 0.26 | 1.79 | 2.79 | 1.58E-06 | 0.00037 |
| **Hypoxia-associated** | | | | | | |
| *Higd1a* | HIG1 hypoxia inducible domain family member 1A | 1.21 | 2.96 | 1.29 | 0.00232 | 0.03304 |
| **Cancer-related signaling pathways** | | | | | | |
| *PI3K/AKT signaling pathway* | | | | | | |
| *Angpt2* | Angiopoietin 2 | 3.91 | 7.65 | 0.97 | 0.00276 | 0.03705 |
| *Il2rb* | Interleukin 2 receptor subunit beta | 1.32 | 3.13 | 1.25 | 0.00028 | 0.00905 |
| *Hsp90aa1* | Heat shock protein 90kDa alpha family class A member 1 | 1.20 | 2.60 | 1.11 | 0.00152 | 0.0259 |
| *RAS signaling pathway* | | | | | | |
| *Rras* | Related RAS viral (R-Ras) oncogene homolog | 13.76 | 29.60 | 1.11 | 4.08E-06 | 0.00069 |
| *Grap* | GRB2-related adaptor protein (RAS) | 4.84 | 9.12 | 0.91 | 0.00091 | 0.01927 |
| *Angpt2* | Angiopoietin 2 | 3.91 | 7.65 | 0.97 | 0.00276 | 0.03705 |
| *Rgs14* | Regulator of G-protein signaling 1 | 3.14 | 5.64 | 0.84 | 0.00231 | 0.03303 |
| *MAPK signaling pathway* | | | | | | |
| *Rras* | Related RAS viral (R-Ras) oncogene homolog | 13.76 | 29.60 | 1.11 | 4.08E-06 | 0.00069 |
| *Trib3* | Tribbles pseudokinase 3 | 7.68 | 12.24 | 0.67 | 0.00292 | 0.03833 |
| *Map3k6* | Mitogen-activated protein kinase kinase kinase 6 | 4.89 | 7.40 | 0.6 | 0.00368 | 0.0436 |
| *Hspa1b* | Heat shock protein family A (Hsp70) member 1B | 1.26 | 3.28 | 1.38 | 4.91E-05 | 0.00313 |
| *Lamtor1* | Late endosomal/lysosomal adaptor, MAPK and MTOR activator 1 | 0.59 | 3.05 | 2.37 | 0.00116 | 0.02209 |
| *Dok4* | Docking protein 4 | 0.69 | 1.84 | 1.42 | 0.00441 | 0.04917 |
| *TGF-b signaling pathway* | | | | | | |
| *Fam89b* | Protein FAM89B | 25.82 | 42.43 | 0.72 | 0.00069 | 0.01608 |
| *Fmod* | Fibromodulin | 0.40 | 1.24 | 1.63 | 0.00344 | 0.04184 |
| *Wnt signaling pathway* | | | | | | |
| *Serpinf1* | Serpin peptidase inhibitor, clade F (alpha-2 antiplasmin, pigment epithelium derived factor), member 1 | 7.48 | 14.38 | 0.94 | 0.00013 | 0.00582 |
| *Wnt8a* | Wnt family member 8A | 1.54 | 4.66 | 1.6 | 0.00011 | 0.00529 |
| *Vangl1* | VANGL planar cell polarity protein 1 | 2.11 | 4.29 | 1.02 | 0.00218 | 0.0325 |
| *Bcl9* | B-Cell CLL/lymphoma 9 | 1.00 | 1.98 | 0.99 | 0.00053 | 0.01355 |
| *Shisa2* | Shisa family member 3 | 0.26 | 1.43 | 2.44 | 8.27E-05 | 0.0044 |
| *JAK-STAT signaling pathway* | | | | | | |
| *Il12rb1* | Interleukin 12 receptor subunit beta 1 | 1.41 | 3.31 | 1.23 | 0.00014 | 0.00606 |
| *Il2rb* | Interleukin 2 receptor subunit beta | 1.32 | 3.13 | 1.25 | 0.00028 | 0.00905 |
| *Calcium signaling pathway* | | | | | | |
| *Tnnc2* | Troponin C2, fast | 25.42 | 41.88 | 0.72 | 0.00204 | 0.03124 |
| *Camk2g* | Calcium/Calmodulin-dependent protein kinase II gamma | 4.66 | 7.63 | 0.71 | 0.00117 | 0.02215 |
| *Tnnc1* | Troponin C type 1 | 0.77 | 5.08 | 2.72 | 0.00164 | 0.02686 |
| **Non-small cell lung cancer** | | | | | | |
| *Erbb2* | Erb-B2 receptor tyrosine kinase 2 | 1.21 | 2.56 | 1.08 | 0.00245 | 0.03404 |
| **Tumor suppressors** | | | | | | |
| *Cyb561d2* | Putative tumor suppressor protein 101F6 | 5.44 | 9.11 | 0.74 | 0.00426 | 0.04818 |
| *Trit1* | tRNA isopentenyltransferase 1 | 3.12 | 6.38 | 1.03 | 0.00031 | 0.00971 |
| *Pdgfrl* | PDGF receptor beta-like tumor suppressor | 0.65 | 2.70 | 2.05 | 0.00062 | 0.01489 |

Genes were sorted by function and Value L_C._
