# Supplementary material for: The systemic tumor response to RNase A treatment affects the expression of genes involved in maintaining cell malignancy
Source: Oncotarget. 2017 Aug 12;8(45):78796–810. doi: 10.18632/oncotarget.20228 (PMC5667999; doi:10.18632/oncotarget.20228)
Supplement: Supplementary file 3 [file oncotarget-08-78796-s003.docx]

**Supplementary Table 2:** Down-regulated genes in tumor of mice with LLC after RNase A treatment (sorted by Value L_C_)

| **Symbol** | **Gene name** | **Value L_C_, RPKM** | **Value L_R_, RPKM** | **Log2 (L_C_/L_R_)** | **p-value** | **q-value** |
| --- | --- | --- | --- | --- | --- | --- |
| **Metabolism related** | | | | | | |
| **Amino sugar and nucleotide sugar metabolism, amino acids and nucleotide metabolism** | | | | | | |
| *Nos2* | Nitric oxide synthase 2, inducible | 11.73 | 7.52 | -0.64 | 0.00063 | 0.01509 |
| *Dguok* | Deoxyguanosine kinase | 16.41 | 6.93 | -1.24 | 5.16E-05 | 0.00319 |
| *Adprm* | CDP-choline phosphohydrolase | 6.44 | 3.37 | -0.93 | 0.00294 | 0.03855 |
| *Twistnb* | TWIST neighbor | 4.51 | 2.05 | -1.14 | 0.00023 | 0.00824 |
| *Setdb2* | Lysine N-methyltransferase 1F | 3.48 | 1.80 | -0.95 | 0.00146 | 0.02569 |
| *Hal* | Histidine ammonia-lyase | 2.87 | 1.11 | -1.37 | 0.00016 | 0.00644 |
| *Amdhd1* | Amidohydrolase domain containing 1 | 2.79 | 0.81 | -1.78 | 3.77E-05 | 0.00274 |
| *Gnpnat1* | Glucosamine-phosphate N-acetyltransferase 1 | 1.41 | 0.26 | -2.45 | 0.00050 | 0.01322 |
| **Carbohydrate metabolism** | | | | | | |
| *Eno3* | Enolase 3 (beta, muscle) | 27.34 | 12.82 | -1.09 | 7.20E-06 | 0.00098 |
| *Galnt3* | Polypeptide N-acetylgalactosaminyltransferase 3 | 13.85 | 6.54 | -1.08 | 2.23E-08 | 1.98E-05 |
| *Alg10b* | Asparagine-linked glycosylation protein 10 homolog B | 10.12 | 5.74 | -0.82 | 5.42E-05 | 0.00330 |
| *Csgalnact2* | Chondroitin sulfate N-acetylgalactosaminyltransferase 2 | 8.60 | 5.68 | -0.60 | 0.00385 | 0.04445 |
| *Alg6* | ALG6, alpha-1,3-glucosyltransferase | 4.31 | 2.47 | -0.80 | 0.00320 | 0.04047 |
| *Ndst2* | Glucosaminyl N-deacetylase/N-sulfotransferase | 4.43 | 2.42 | -0.87 | 0.00061 | 0.01489 |
| *Gxylt1* | Glucoside xylosyltransferase 1 | 3.86 | 2.18 | -0.83 | 0.00024 | 0.00839 |
| *Xylt1* | Xylosyltransferase I | 1.25 | 0.42 | -1.58 | 0.00285 | 0.03775 |
| **Cytochrome P450-associated metabolism** | | | | | | |
| *Hsd11b1* | Hydroxysteroid (11-beta) dehydrogenase | 4.45 | 1.92 | -1.21 | 0.00280 | 0.03745 |
| *Cyp27b1* | Cytochrome P450, family 27, subfamily B, polypeptide 1 | 3.27 | 1.62 | -1.01 | 0.00263 | 0.03584 |
| *Cyp26b1* | Cytochrome P450, family 26, subfamily B, polypeptide 1 | 1.22 | 0.27 | -2.20 | 4.08E-05 | 0.00280 |
| **Inositolphosphate** | | | | | | |
| *Pi4k2b* | Phosphatidylinositol 4-kinase type 2 beta | 31.67 | 22.66 | -0.48 | 0.00374 | 0.04384 |
| **Metabolism of lipids and fatty acids** | | | | | | |
| *Agpat2* | 1-Acylglycerol-3-phosphate O-acyltransferase 2 | 26.46 | 14.39 | -0.89 | 0.00049 | 0.01309 |
| *Cd5l* | CD5 molecule-like | 10.16 | 4.57 | -1.15 | 5.47E-06 | 0.00087 |
| *Mcat* | Mitochondrial malonyltransferase | 7.60 | 4.17 | -0.87 | 0.00149 | 0.02575 |
| *Elovl6* | ELOVL fatty acid elongase 6 | 6.49 | 3.36 | -0.95 | 3.02E-06 | 0.00058 |
| *Lipt1* | Lipoyltransferase 1 | 4.78 | 2.33 | -1.04 | 0.00337 | 0.04165 |
| *B4galt6* | Beta-1,4-galactosyltransferase 6 | 2.34 | 1.12 | -1.06 | 0.00013 | 0.00582 |
| *B3galnt1* | Globoside synthase | 1.71 | 0.26 | -2.69 | 0.00080 | 0.01775 |
| **Oxidative phosphorylation** | | | | | | |
| *Cox7b* | Cytochrome C oxidase subunit VIIb | 38.69 | 19.50 | -0.99 | 4.08E-06 | 0.00070 |
| *Ndufa5* | NADH dehydrogenase (ubiquinone) 1 alpha subcomplex, 5 | 29.21 | 16.40 | -0.83 | 0.00435 | 0.04875 |
| *Atp8b4* | ATPase, class I, type 8B, member 4 | 1.80 | 0.73 | -1.30 | 4.48E-05 | 0.00298 |
| **Nicotinate and nicotinamide metabolism** | | | | | | |
| *Bst1* | Bone marrow stromal cell antigen 1 | 10.19 | 4.78 | -1.09 | 5.47E-06 | 0.00087 |
| **Glutathione metabolism** | | | | | | |
| *Gclm* | Glutamate-cysteine ligase, modifier subunit | 19.43 | 12.17 | -0.67 | 0.00222 | 0.03272 |
| *Gss* | Glutathione synthetase | 7.18 | 3.89 | -0.88 | 0.00162 | 0.02674 |
| *Gpx7* | Glutathione peroxidase 7 | 2.20 | 0.30 | -2.89 | 0.00300 | 0.03875 |
| **Folate metabolism** | | | | | | |
| *Dhfr* | Dihydrofolate reductase | 9.83 | 6.54 | -0.59 | 0.00360 | 0.04319 |
| *Mthfd2l* | Methylenetetrahydrofolate dehydrogenase (NADP+ dependent) 2-like | 1.90 | 0.66 | -1.52 | 0.00241 | 0.03383 |
| **Cancer related** | | | | | | |
| **Angiogenesis** | | | | | | |
| *Cxcl5* | Chemokine (C-X-C motif) ligand 5 | 5.32 | 2.37 | -1.17 | 0.00057 | 0.01409 |
| *Filip1l* | Filamin A interacting protein 1-like | 4.18 | 2.11 | -0.98 | 0.00424 | 0.04799 |
| *Smoc2* | Secreted modular calcium-binding protein 2 | 2.79 | 1.37 | -1.03 | 0.00219 | 0.03251 |
| *Angptl1* | Angiopoietin-like 1 | 1.21 | 0.15 | -3.01 | 0.00445 | 0.04935 |
| **Apoptosis** | | | | | |  |
| *Lcn2* | Lipocalin 2 (Oncogene 24p3) | 58.01 | 34.52 | -0.75 | 0.00016 | 0.00643 |
| *Ctsc* | Cathepsin C | 37.67 | 26.68 | -0.50 | 0.00284 | 0.03775 |
| *Gzmb* | Cathepsin G-Like | 9.07 | 2.59 | -1.81 | 7.68E-08 | 4.09E-05 |
| *Hipk3* | Homeodomain interacting protein kinase 3 | 8.96 | 6.27 | -0.51 | 0.00374 | 0.04384 |
| *Phlda1* | Apoptosis-associated nuclear protein | 7.17 | 3.00 | -1.26 | 1.32E-05 | 0.00149 |
| *Bcl2l2* | BCL2-like | 4.95 | 2.71 | -0.87 | 0.00067 | 0.01571 |
| *Casp9* | Caspase 9, apoptosis-related cysteine peptidas | 4.68 | 2.43 | -0.94 | 0.00025 | 0.00849 |
| *Ctso* | Cathepsin O | 2.44 | 0.92 | -1.40 | 7.07E-05 | 0.00394 |
| *Casp12* | Caspase 12 | 2.21 | 0.81 | -1.45 | 0.00066 | 0.01571 |
| *Coro2a* | Coronin, actin binding protein, 2A | 1.31 | 0.33 | -2.01 | 0.00018 | 0.00710 |
| **Cell adhesion, migration, invasion** | | | | | | |
| *S100a4* | Metastasin | 72.78 | 42.34 | -0.78 | 0.00074 | 0.01684 |
| *Col1a1* | Collagen, Type I, Alpha 1 | 55.80 | 35.41 | -0.66 | 0.00018 | 0.00689 |
| *Cav2* | Caveolin 2 | 54.16 | 37.09 | -0.55 | 0.00092 | 0.01947 |
| *Rab1* | RAB1A, member RAS oncogene family | 46.64 | 33.27 | -0.49 | 0.00310 | 0.03979 |
| *Thbs1* | Thrombospondin 1 | 30.29 | 20.16 | -0.59 | 0.00045 | 0.01237 |
| *Rasgrf1* | Ras protein-specific guanine nucleotide-releasing factor 1 | 29.90 | 10.33 | -1.53 | 1.11E-09 | 2.35E-06 |
| *Shc4* | SHC (Src homology 2 domain containing) family, member 4 | 8.01 | 0.91 | -3.14 | 6.86E-14 | 3.65E-10 |
| *Lrg1* | Leucine-rich alpha-2-glycoprotein | 5.37 | 2.60 | -1.04 | 0.00393 | 0.04504 |
| *Cxcl5* | Chemokine (C-X-C motif) ligand 5 | 5.32 | 2.37 | -1.17 | 0.00057 | 0.01409 |
| *Itga7* | Integrin, alpha 7 | 3.72 | 1.53 | -1.28 | 6.95E-06 | 0.00097 |
| *Smoc2* | Secreted modular calcium-binding protein 2 | 2.79 | 1.37 | -1.03 | 0.00219 | 0.03251 |
| *Ccnd2* | Cyclin D2 | 1.63 | 0.59 | -1.46 | 1.89E-05 | 0.00188 |
| **Cell cycle control,** **transformation** | | | | | | |
| *S100a9* | Calgranulin-B | 269.93 | 188.42 | -0.52 | 0.00209 | 0.03155 |
| *Nedd8* | Neddylin | 83.75 | 46.05 | -0.86 | 3.01E-05 | 0.00239 |
| *Incenp* | Inner centromere protein antigens 135/155kDa | 13.08 | 8.20 | -0.67 | 0.00053 | 0.01355 |
| *Cenpw* | Cancer-up-regulated gene 2 protein | 4.13 | 1.83 | -1.17 | 0.00297 | 0.03870 |
| *Nudt6* | Antisense basic fibroblast growth factor | 4.44 | 1.21 | -1.88 | 0.00064 | 0.01526 |
| **Signaling pathways** | | | | | | |
| *PI3K/AKT signaling pathway* | | | | | | |
| *Col1a1* | Collagen, Type I, Alpha 1 | 55.80 | 35.41 | -0.66 | 0.00018 | 0.00689 |
| *Thbs1* | Thrombospondin 1 | 30.29 | 20.16 | -0.59 | 0.00045 | 0.01237 |
| *Il4ra* | Interleukin 4 receptor | 20.39 | 14.71 | -0.47 | 0.00436 | 0.04880 |
| *Csf3* | Colony stimulating factor 3 (granulocyte) | 9.32 | 3.87 | -1.27 | 2.87E-05 | 0.00234 |
| *Jak2* | Janus kinase 2 | 8.78 | 5.24 | -0.74 | 0.00120 | 0.02258 |
| *Tnc* | Tenascin C | 6.86 | 4.59 | -0.58 | 0.00156 | 0.02626 |
| *Jak3* | Janus kinase 3 | 4.25 | 1.88 | -1.18 | 1.93E-05 | 0.00188 |
| *Itga7* | Integrin, alpha 7 | 3.72 | 1.53 | -1.28 | 6.95E-06 | 0.00097 |
| *Csf3r* | CD114 antigen | 2.16 | 0.93 | -1.22 | 0.00045 | 0.01241 |
| *Il7r* | Interleukin 7 receptor | 1.86 | 0.45 | -2.05 | 2.37E-05 | 0.00207 |
| *Ccnd2* | Cyclin D2 | 1.63 | 0.59 | -1.46 | 1.89E-05 | 0.00188 |
| *Itga4* | Integrin Subunit Alpha 4 | 1.33 | 0.75 | -0.83 | 0.00257 | 0.03522 |
| *RAS signaling pathway* | | | | | | |
| *Rasgrf1* | Ras protein-specific guanine nucleotide-releasing factor 1 | 29.90 | 10.33 | -1.53 | 1.11E-09 | 2.35E-06 |
| *Kras* | Kirsten rat sarcoma viral oncogene homolog | 18.49 | 13.00 | -0.51 | 0.00231 | 0.03303 |
| *Shc4* | SHC (Src homology 2 domain containing) family, member 4 | 8.01 | 0.91 | -3.14 | 6.86E-14 | 3.65E-10 |
| *Pld1* | Choline phosphatase 1 | 1.88 | 0.63 | -1.59 | 1.04E-05 | 0.00123 |
| *MAPK signaling pathway* | | | | | | |
| *Ccl7* | Monocyte chemoattractant protein 3 | 89.78 | 42.91 | -1.07 | 1.56E-08 | 1.67E-05 |
| *Dusp6* | Dual specificity phosphatase 6 | 76.41 | 50.37 | -0.60 | 0.00040 | 0.01164 |
| *Il1b* | Interleukin 1, beta | 61.23 | 38.23 | -0.68 | 8.56E-05 | 0.00442 |
| *Cd14* | Cd14 molecule | 32.23 | 19.21 | -0.75 | 0.00011 | 0.00523 |
| *Rasgrf1* | Ras protein-specific guanine nucleotide-releasing factor 1 | 29.90 | 10.33 | -1.53 | 1.11E-09 | 2.35E-06 |
| *Stk24* | Serine/threonine kinase 24 | 16.21 | 10.17 | -0.67 | 0.00058 | 0.01432 |
| *Il1r2* | Interleukin 1 receptor, type II | 10.66 | 5.51 | -0.95 | 0.00074 | 0.01684 |
| *Ccl5* | Chemokine (C-C motif) ligand 5 | 10.56 | 3.19 | -1.73 | 0.00192 | 0.02980 |
| *Map2k4* | Mitogen-activated protein kinase kinase 4 | 6.70 | 4.12 | -0.70 | 0.00184 | 0.02888 |
| *Hspa2* | Heat shock 70kDa protein 2 | 5.88 | 2.73 | -1.11 | 5.93E-05 | 0.00357 |
| *Itgax* | CD11C | 1.42 | 0.47 | -1.59 | 0.00028 | 0.00905 |
| *TGF-b signaling pathway* | | | | | | |
| *Ccl7* | Monocyte chemoattractant protein 3 | 89.78 | 42.91 | -1.07 | 1.56E-08 | 1.67E-05 |
| *Thbs1* | Thrombospondin 1 | 30.29 | 20.16 | -0.59 | 0.00045 | 0.01237 |
| *Ccl5* | Chemokine (C-C motif) ligand 5 | 10.56 | 3.19 | -1.73 | 0.00192 | 0.02980 |
| *Acvr1b* | Activin A receptor, type IB | 5.06 | 2.63 | -0.94 | 0.00029 | 0.00939 |
| *Rbx1* | Ring-Box 1, E3 ubiquitin protein ligase | 3.13 | 0.52 | -2.60 | 3.29E-05 | 0.00249 |
| *Fst* | Follistatin | 1.22 | 0.14 | -3.12 | 0.00122 | 0.02287 |
| *Wnt signaling pathway* | | | | | | |
| *Chd8* | Chromodomain helicase DNA binding protein 8 | 10.71 | 7.09 | -0.59 | 0.00044 | 0.01229 |
| *Lrrfip2* | LRR binding FLII interacting protein 2 | 10.20 | 6.72 | -0.60 | 0.00344 | 0.04184 |
| *Dkk2* | Dickkopf WNT signaling pathway inhibitor 2 | 8.50 | 5.09 | -0.74 | 0.00046 | 0.01241 |
| *Hmgxb4* | HMG box domain containing 4 | 3.26 | 1.61 | -1.02 | 0.00033 | 0.01009 |
| *Rbx1* | Ring-Box 1, E3 ubiquitin protein ligase | 3.13 | 0.52 | -2.60 | 3.29E-05 | 0.00249 |
| *Lgr4* | Leucine-rich repeat containing G protein-coupled receptor 4 | 2.84 | 1.67 | -0.77 | 0.00346 | 0.04197 |
| *Ccnd2* | Cyclin D2 | 1.63 | 0.59 | -1.46 | 1.89E-05 | 0.00188 |
| *JAK-STAT pathway* | | | | | | |
| *Ccl7* | Monocyte chemoattractant protein 3 | 89.78 | 42.91 | -1.07 | 1.56E-08 | 1.67E-05 |
| *Il4ra* | Interleukin 4 receptor | 20.39 | 14.71 | -0.47 | 0.00436 | 0.04880 |
| *Shcbp1* | Protein expressed in activated lymphocytes | 16.22 | 9.86 | -0.72 | 0.00054 | 0.01356 |
| *Ccl5* | Chemokine (C-C motif) ligand 5 | 10.56 | 3.19 | -1.73 | 0.00192 | 0.02980 |
| *Csf3* | Colony stimulating factor 3 (granulocyte) | 9.32 | 3.87 | -1.27 | 2.87E-05 | 0.00234 |
| *Jak2* | Janus kinase 2 | 8.78 | 5.24 | -0.74 | 0.00120 | 0.02258 |
| *Jak3* | Janus kinase 3 | 4.25 | 1.88 | -1.18 | 1.93E-05 | 0.00188 |
| *Csf3r* | CD114 antigen | 2.16 | 0.93 | -1.22 | 0.00045 | 0.01241 |
| *Il21r* | Interleukin 21 receptor | 1.93 | 0.69 | -1.49 | 0.00109 | 0.02143 |
| *Il7r* | Interleukin 7 receptor | 1.86 | 0.45 | -2.05 | 2.37E-05 | 0.00207 |
| *Ccnd2* | Cyclin D2 | 1.63 | 0.59 | -1.46 | 1.89E-05 | 0.00188 |
| *Calcium signaling pathway* | | | | | | |
| *P2rx7* | Purinergic receptor P2X, ligand gated ion channel, 7 | 7.45 | 2.82 | -1.40 | 0.00100 | 0.02030 |
| *Tnc* | Tenascin C | 6.86 | 4.59 | -0.58 | 0.00156 | 0.02626 |
| **Cancer-associated^a^** | | | | | | |
| *Orai1* | ORAI calcium release-activated calcium modulator | 13.60 | 6.73 | -1.01 | 1.59E-05 | 0.00166 |
| *Dpp3* | Dipeptidyl-peptidase 3 | 24.46 | 17.34 | -0.50 | 0.00431 | 0.04856 |
| *Arhgef1* | Rho guanine nucleotide exchange factor (GEF) 1 | 23.04 | 15.28 | -0.59 | 0.00069 | 0.01611 |
| *Steap1* | Six transmembrane epithelial antigen of the prostate 1 | 19.82 | 11.67 | -0.76 | 0.00123 | 0.02290 |
| *Arhgef11* | Rho guanine nucleotide exchange factor (GEF) 11 | 10.10 | 5.77 | -0.81 | 1.18E-05 | 0.00135 |
| *Skp2* | S-phase kinase-associated protein 2, E3 ubiquitin protein ligase | 8.89 | 5.29 | -0.75 | 0.00097 | 0.01984 |
| *Tpd52* | Tumor protein D5 | 9.46 | 4.87 | -0.96 | 9.17E-05 | 0.00457 |
| *Mllt11* | Myeloid/Lymphoid or mixed-lineage leukemia; translocated to, 11 | 3.39 | 0.97 | -1.80 | 4.11E-06 | 0.00070 |
| *Laptm4b* | Lysosomal protein transmembrane 4 beta | 2.51 | 0.97 | -1.37 | 0.00298 | 0.03875 |
| *Rfng* | RFNG O-fucosylpeptide 3-beta-N-acetylglucosaminyltransferase | 2.47 | 0.94 | -1.40 | 0.00158 | 0.02648 |
| *Ehbp1* | NPF calponin-like protein | 1.74 | 0.85 | -1.04 | 0.00313 | 0.04002 |
| *Rbm6* | Lung cancer antigen NY-LU-12 | 1.40 | 0.44 | -1.69 | 0.00081 | 0.01775 |
| **Non-small cell lung cancer** | | | | | | |
| *Jak3* | Janus kinase 3 | 4.25 | 1.88 | -1.18 | 1.93E-05 | 0.00188 |
| **MicroRNA in cancer** | | | | | | |
| *Ezh2* | Enhancer of Zeste 2 polycomb repressive complex 2 subunit | 27.39 | 15.17 | -0.85 | 3.19E-06 | 0.00060 |
| *Lin28a* | Lin-28 homolog A | 15.71 | 8.78 | -0.84 | 5.95E-06 | 0.00087 |
| *Zcchc6* | Zinc finger, CCHC domain containing 6 | 10.04 | 6.04 | -0.73 | 6.89E-05 | 0.00393 |
| *Tnrc6a* | Trinucleotide repeat containing 6A | 6.30 | 4.22 | -0.58 | 0.00130 | 0.02375 |
| *Zcchc11* | TUTase | 4.00 | 2.48 | -0.69 | 0.00222 | 0.03269 |
| **Tumor suppressors** | | | | | | |
| *Armcx1* | Armadillo repeat containing, X-linked 1 | 5.26 | 2.24 | -1.23 | 0.00016 | 0.00644 |
| *Brca2* | BRCA2, DNA repair associated | 2.83 | 1.48 | -0.93 | 0.00016 | 0.00644 |
| *Tssc1* | Tumor suppressing subtransferable candidate 1 | 2.07 | 0.50 | -2.04 | 0.00151 | 0.02586 |
| *Scai* | Suppressor of cancer cell invasion | 1.39 | 0.81 | -0.77 | 0.00362 | 0.04321 |

Genes were sorted by function and Value L_C._

^a^Expression of these genes is up-regulated in multiple cancer cell lines.
