# Supplementary material for: The systemic tumor response to RNase A treatment affects the expression of genes involved in maintaining cell malignancy
Source: Oncotarget. 2017 Aug 12;8(45):78796–810. doi: 10.18632/oncotarget.20228 (PMC5667999; doi:10.18632/oncotarget.20228)
Supplement: Supplementary file 4 [file oncotarget-08-78796-s004.docx]

**Supplementary Table 3:** Upregulated transcription-related genes in tumor of mice with LLC after RNase A treatment (sorted by Value L_R_)

| **Gene** | **Function** | **Value L_C_, RPKM** | **Value L_R_, RPKM** | **Log2 (L_R_/L_C_)** | **p-value** | **q-value** |
| --- | --- | --- | --- | --- | --- | --- |
| *Pmf1* | Transcriptional activators and co-activators | 27.88 | 45.89 | 0.72 | 0.00031 | 0.00970 |
| *Klf9* |  | 5.93 | 9.45 | 0.67 | 0.00151 | 0.02586 |
| *Bcas3* |  | 1.83 | 3.52 | 0.94 | 0.00246 | 0.03418 |
| *Zfp513* | Transcriptional regulators | 3.29 | 5.99 | 0.86 | 0.00206 | 0.03139 |
| *Zfp296* |  | 1.60 | 3.72 | 1.22 | 0.00335 | 0.04165 |
| *Zfp790* |  | 2.06 | 3.61 | 0.81 | 0.00174 | 0.02796 |
| *Zfp651* |  | 1.71 | 2.83 | 0.72 | 0.00317 | 0.04026 |
| *Zfp316* |  | 0.59 | 1.68 | 1.50 | 1.24E-06 | 0.0003 |
| *Mbd1* | Transcriptional repressors and co-repressors | 6.10 | 10.07 | 0.72 | 0.00108 | 0.02126 |
| *Pcgf5* |  | 5.40 | 9.98 | 0.89 | 0.00114 | 0.02193 |
| *Nelf* |  | 5.61 | 9.80 | 0.80 | 0.00098 | 0.02002 |
| *Cbfa2t2* |  | 3.40 | 6.20 | 0.86 | 3.20E-05 | 0.00247 |
| *Per1* |  | 3.75 | 6.04 | 0.69 | 0.00145 | 0.02565 |
| *Gtf2h2* | Transcriptional factors | 15.21 | 25.89 | 0.77 | 9.74E-05 | 0.00480 |
| *E2f1* |  | 7.3 | 12.28 | 0.74 | 0.00059 | 0.01442 |
| *Gabpb1* |  | 4.14 | 8.16 | 0.98 | 0.00041 | 0.01178 |
| *Klf5* |  | 2.85 | 6.59 | 1.21 | 8.55E-05 | 0.00442 |
| *Zfp691* |  | 1.05 | 4.88 | 2.22 | 6.19E-05 | 0.00368 |
| *Hoxc10* |  | 1.86 | 4.47 | 1.26 | 0.00028 | 0.00905 |
| *Gas7* |  | 2.48 | 4.13 | 0.74 | 0.00129 | 0.02375 |
| *Tfeb* |  | 0.93 | 4.13 | 2.14 | 1.08E-06 | 0.00027 |
| *Hes6* |  | 1.62 | 4.10 | 1.34 | 0.00231 | 0.03303 |
| *Ovol1* |  | 1.07 | 2.56 | 1.25 | 0.00055 | 0.01385 |
| *Hnf1b* |  | 0.69 | 2.13 | 1.63 | 0.00022 | 0.00812 |
| *Zscan10* |  | 0.12 | 1.98 | 4.09 | 1.96E-05 | 0.00188 |
| *Hoxb8* |  | 0.40 | 1.53 | 1.96 | 0.00062 | 0.01489 |
| *Cc2d1a* |  | 0.50 | 1.51 | 1.68 | 0.00045 | 0.01241 |
| *Zfp167* |  | 0.24 | 1.33 | 2.48 | 0.00151 | 0.02586 |
| *Pou2f3* |  | 0.12 | 1.23 | 3.30 | 0.00073 | 0.01671 |
