# Supplementary material for: The systemic tumor response to RNase A treatment affects the expression of genes involved in maintaining cell malignancy
Source: Oncotarget. 2017 Aug 12;8(45):78796–810. doi: 10.18632/oncotarget.20228 (PMC5667999; doi:10.18632/oncotarget.20228)
Supplement: Supplementary file 5 [file oncotarget-08-78796-s005.docx]

**Supplementary Table 4:** Down-regulated transcription-related genes in tumor of mice with LLC after RNase A treatment (sorted by Value L_C_)

| **Gene** | **Function** | **Value L_C_, RPKM** | **Value L_R_, RPKM** | **Log2 (L_R_/L_C_)** | **p-value** | **q-value** |
| --- | --- | --- | --- | --- | --- | --- |
| *Bcl9l* | Transcriptional activators and coactivators | 3.37 | 1.46 | -1.21 | 3.76E-06 | 0.00067 |
| *Zmiz2* |  | 2.91 | 1.44 | -1.02 | 0.00187 | 0.02923 |
| *Hipk2* |  | 2.82 | 1.12 | -1.33 | 0.00022 | 0.00801 |
| *Znrd1* | Transcription regulator | 7.98 | 2.79 | -1.52 | 0.00241 | 0.03383 |
| *Sertad2* |  | 7.51 | 4.76 | -0.66 | 0.00171 | 0.02762 |
| *Zfp292* |  | 7.33 | 4.94 | -0.57 | 0.00085 | 0.01824 |
| *Tfe3* |  | 6.59 | 2.97 | -1.15 | 0.00035 | 0.01047 |
| *Chd7* |  | 5.15 | 3.34 | -0.62 | 0.00074 | 0.01684 |
| *Zfp81* |  | 5.14 | 2.34 | -1.13 | 0.00016 | 0.00643 |
| *Rfx5* |  | 2.86 | 1.13 | -1.34 | 8.27E-06 | 0.00103 |
| *Med21* |  | 58.00 | 37.32 | -0.64 | 0.00168 | 0.02737 |
| *Med6* |  | 12.53 | 5.98 | -1.07 | 0.00041 | 0.01178 |
| *Zbtb6* |  | 10.50 | 7.02 | -0.58 | 0.00176 | 0.02807 |
| *Bmp2k* |  | 10.37 | 7.02 | -0.56 | 0.00118 | 0.02236 |
| *Zfp948* |  | 8.67 | 5.13 | -0.76 | 0.00120 | 0.02252 |
| *Esf1* |  | 8.66 | 5.36 | -0.69 | 0.00119 | 0.02244 |
| *Zkscan1* |  | 8.28 | 4.89 | -0.76 | 0.00018 | 0.00710 |
| *Mll1* |  | 7.18 | 5.15 | -0.48 | 0.00358 | 0.04306 |
| *Pias1* |  | 5.00 | 2.83 | -0.82 | 0.00079 | 0.01761 |
| *Kdm6a* |  | 2.50 | 1.18 | -1.08 | 0.00013 | 0.00577 |
| *Zfp7* |  | 2.46 | 1.17 | -1.07 | 0.00317 | 0.04026 |
| *Zfp597* |  | 2.12 | 0.91 | -1.22 | 3.78E-05 | 0.00274 |
| *Zbtb24* |  | 1.95 | 0.86 | -1.19 | 0.00345 | 0.04184 |
| *Prdm10* |  | 1.93 | 0.89 | -1.12 | 0.00238 | 0.03364 |
| *Pak6* |  | 1.90 | 0.39 | -1.72 | 0.00180 | 0.02853 |
| *Kdm5b* |  | 1.89 | 1.01 | -0.90 | 0.00144 | 0.02550 |
| *Spint1* |  | 1.77 | 0.58 | -1.61 | 0.00216 | 0.03229 |
| *Thap7* | Transcriptional repressors and co-repressors | 7.63 | 3.12 | -1.29 | 0.00028 | 0.00905 |
| *Mxd4* |  | 7.02 | 3.37 | -1.06 | 0.00235 | 0.03326 |
| *Tle1* |  | 5.06 | 3.10 | -0.10 | 0.00220 | 0.03265 |
| *Sfmbt1* |  | 3.07 | 1.90 | -0.69 | 0.00362 | 0.04321 |
| *Gtf2h5* | Transcriptional factors | 17.35 | 10.52 | -0.72 | 0.00352 | 0.04258 |
| *Tceb1* |  | 14.37 | 5.37 | -1.42 | 4.51E-06 | 0.00074 |
| *Ccnc* |  | 13.60 | 6.94 | -0.97 | 6.55E-05 | 0.00379 |
| *Top1* |  | 12.25 | 7.94 | -0.63 | 0.00084 | 0.01808 |
| *Adnp* |  | 10.85 | 7.49 | -0.53 | 0.00301 | 0.03888 |
| *Ccnt2* |  | 7.21 | 4.39 | -0.72 | 0.00138 | 0.02472 |
| *Iws1* |  | 4.38 | 2.42 | -0.86 | 1.22E-05 | 0.00138 |
| *Atf7* |  | 3.28 | 1.53 | -1.10 | 0.00050 | 0.01322 |
| *Dlx6* |  | 3.00 | 0.34 | -3.12 | 0.00297 | 0.03870 |
| *Rhox2d* |  | 2.61 | 0.00 | - | 0.00017 | 0.00672 |
| *Gpbp1l1* |  | 2.30 | 0.67 | -1.77 | 8.38E-06 | 0.00103 |
| *Akna* |  | 2.18 | 1.19 | -0.87 | 0.00209 | 0.03155 |
| *Dlx1* |  | 1.69 | 0.51 | -1.73 | 0.00291 | 0.03823 |
